# Synergistic Mechanism of Sub‐Nanometric Ru Clusters Anchored on Tungsten Oxide Nanowires for High‐Efficient Bifunctional Hydrogen Electrocatalysis

**DOI:** 10.1002/advs.202206096

**Published:** 2023-01-03

**Authors:** Yecan Pi, Ziming Qiu, Yi Sun, Hirofumi Ishii, Yen‐Fa Liao, Xiuyun Zhang, Han‐Yi Chen, Huan Pang

**Affiliations:** ^1^ School of Chemistry and Chemical Engineering Yangzhou University Jiangsu 225002 China; ^2^ Key Laboratory of Advanced Energy Materials Chemistry (Ministry of Education) College of Chemistry Nankai University Tianjin 300071 China; ^3^ National Synchrotron Radiation Research Center 101 Hsin‐Ann Road, Hsinchu Science Park Hsinchu 30076 Taiwan; ^4^ Department of Materials Science and Engineering National Tsing Hua University 101, Sec. 2, Kuang‐Fu Road Hsinchu 300044 Taiwan

**Keywords:** hydrogen electrocatalysis, metal‐support interaction, nanowires, ruthenium, sub‐nanometric cluster

## Abstract

The construction of strong interactions and synergistic effects between small metal clusters and supports offers a great opportunity to achieve high‐performance and cost‐effective heterogeneous catalysis, however, studies on its applications in electrocatalysis are still insufficient. Herein, it is reported that W_18_O_49_ nanowires supported sub‐nanometric Ru clusters (denoted as Ru SNC/W_18_O_49_ NWs) constitute an efficient bifunctional electrocatalyst for hydrogen evolution/oxidation reactions (HER and HOR) under acidic condition. Microstructural analyses, X‐ray absorption spectroscopy, and density functional theory (DFT) calculations reveal that the Ru SNCs with an average Ru—Ru coordination number of 4.9 are anchored to the W_18_O_49_ NWs via Ru—O—W bonds at the interface. The strong metal‐support interaction leads to the electron‐deficient state of Ru SNCs, which enables a modulated Ru—H strength. Furthermore, the unique proton transport capability of the W_18_O_49_ also provides a potential migration channel for the reaction intermediates. These components collectively enable the remarkable performance of Ru SNC/W_18_O_49_ NWs for hydrogen electrocatalysis with 2.5 times of exchange current density than that of carbon‐supported Ru nanoparticles, and even rival the state‐of‐the‐art Pt catalyst. This work provides a new prospect for the development of supported sub‐nanometric metal clusters for efficient electrocatalysis.

## Graphical abstract

Sub‐nanometric Ru clusters loaded with W_18_O_49_ NWs are successfully prepared via a facile one‐pot hydrothermal method. Combined characterizations and theoretical calculations reveal that the strong electronic metal‐support interaction between Ru clusters and W_18_O_49_ optimizes the binding strength of hydrogen intermediate at Ru sites, which exhibits attractive performance for hydrogen electrocatalysis that rival the state‐of‐the‐art Pt.

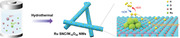

## 1 Introduction

Dispersion of active metal nanoparticles (NPs) on supports is an effective strategy to improve the utilization of precious metals and thus reduce catalyst costs, which has been widely used in the design of heterogeneous catalysts.^[^ ^1^, ^2^, ^3^ ^]^ Extensive studies have shown that as the size of NPs decreases to the sub‐nanometer scale (<50 atoms), the ratio of the corresponding surface to bulk atoms (metal dispersion) will increase greatly, which is significant for the construction of cost‐effective catalysts.^[^ ^4^, ^5^, ^6^ ^]^ In addition, compared to large NPs, the specifically lower average metal‐metal coordination number in sub‐nanometric clusters (SNCs) often results in a dramatic difference in the adsorption and conversion with reactant molecules.^[^ ^7^, ^8^, ^9^, ^10^ ^]^ On the other hand, when in contact with the support, the SNCs also exhibit stronger metal‐support interactions due to the higher proportion of interface sites, which not only contribute to enhanced stability, but also further modulate the electronic structure of the metal and thus influence its catalytic properties.^[^ ^11^, ^12^ ^]^ It should be noted that, unlike the single‐atom catalysts (SACs) using isolated metal atoms as the active center, an important feature of SNCs is that they have metal‐metal bonds. Such adjacent metal sites can provide more opportunities for the adsorption and activation of reactant molecules and thus have broader applicability for different reactions.^[^ ^13^, ^14^ ^]^

However, although the carbon‐based supports commonly used in electrocatalysts have good electrical conductivity, the interaction between the metal NPs and the support is usually weak, and the supported NPs often aggregate or fall off from the carbon substrates and thus cause catalyst failure.^[^ ^15^, ^16^, ^17^, ^18^ ^]^ Moreover, the inherent inertness of carbon usually makes it difficult to participate directly in the electrocatalytic reactions and thus limits their ability to play a more important synergistic role during electrocatalysis.^[^ ^19^, ^20^ ^]^ In contrast, metal oxides are a promising class of supports in which metal NPs can be anchored on the supports via metal‐oxygen bonds, forming strong electronic metal‐support interaction (EMSI).^[^ ^21^, ^22^, ^23^ ^]^ Particularly for the reducible metal oxides (e.g., TiO_2‐*δ*_, CeO_2‐*δ*_, and WO_3‐*δ*_), the presence of coordinatively unsaturated cationic sites in these oxides not only facilitate electron transport in the oxide network, but also provide a promising way to promote the unique synergistic effect during the catalytic process.^[^ ^24^, ^25^, ^26^ ^]^ For example, the migration of hydrogen atoms from metal NPs to reducible metal oxide supports (hydrogen spillover) has been widely reported to play a key facilitating role in many heterogeneous catalysis applications. However, despite significant efforts have been made to develop NPs/oxide hybrid materials over recent years, there is still a lack of facile synthetic methods to construct the complexes of metal SNCs and oxide supports. In addition, compared with the extensive attention to the mechanism of metal‐support interactions in thermal catalysis, more efforts are also necessary to provide deeper investigation regarding the synergistic mechanism between metal SNCs and oxide supports under specific electrocatalytic reactions.^[^ ^27^, ^28^ ^]^

Herein, we report a facile one‐pot hydrothermal method to prepare the tungsten oxide nanowires supported Ru sub‐nanometric clusters (Ru SNC/W_18_O_49_ NWs), which exhibit apparent electrocatalytic performance for both hydrogen evolution and oxidation reactions (HER and HOR). Particularly, the Ru SNC/W_18_O_49_ NWs only require an overpotential of 21 mV to drive HER current densities of 10 mA cm^−2^, with a Tafel slope of 35 mV dec^−1^ and exchange current density of 2.5 mA cm^−2^, outperforming the comparative Ru NPs and most of the other reported catalysts (Table S3, Supporting Information). In addition, the Ru SNC/W_18_O_49_ NWs also exhibits attractive HOR activity, and better tolerance to the CO and O_2_ impurities compared to the commercial Pt/C. Microstructural analyses, X‐ray absorption spectroscopy, and theoretical calculations together revealed that Ru SNCs are anchored on the W_18_O_49_ support through the Ru—O—W bonds, which resulted in the electrons transfer from Ru to the W_18_O_49_ support. Such EMSI not only helped to stabilize Ru SNC, but also regulate the electronic structure of Ru and thus optimized the binding strength of the hydrogen intermediate. Furthermore, the hydrogen spillover effect of W_18_O_49_ support facilitates reaction kinetics by providing the hydrogen intermediate transfer pathway. The outstanding catalytic properties coupled with relatively low cost make Ru SNC/W_18_O_49_ NWs the highly promising HER/HOR catalyst to replace Pt.

## 2 Results and Discussion

**Figure 1:**
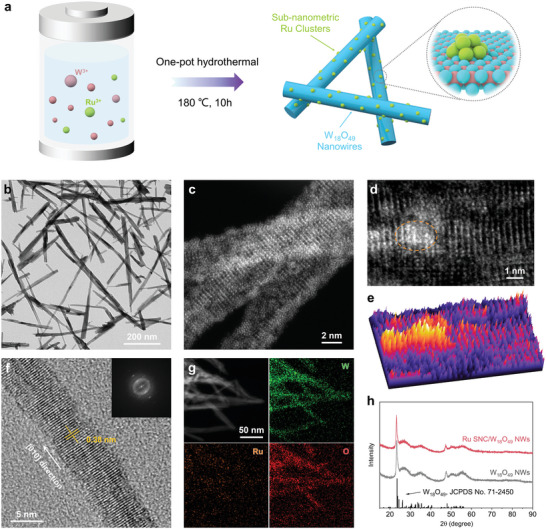
a) Schematic illustration for the synthesis of Ru SNC/W_18_O_49_ NWs. b) TEM and c,d) HAADF‐STEM images of Ru SNC/W_18_O_49_ NWs. e) Corresponding 3D HAADF‐STEM surface plot image of (d). f) HRTEM image (inset shows the corresponding fast Fourier transform pattern), g) STEM‐EDS elemental mappings of Ru SNC/W_18_O_49_ NWs. h) XRD patterns of Ru SNC/W_18_O_49_ NWs and W_18_O_49_ NWs.

The Ru SNC/W_18_O_49_ NWs were synthesized via a facile one‐pot hydrothermal method using tungsten (VI) chloride (WCl_6_), ruthenium (III) chloride (RuCl_3_), and ethanol as metal precursor and solvent, respectively (**Figure** **1**a). Transmission electron microscopy (TEM) images show that the obtained Ru SNC/W_18_O_49_ NWs have a mean diameter of 10.8 ± 3.4 nm and a length extending to several hundreds of nanometers (Figure 1b; Figures S1 and S2, Ru‐O‐W). No obvious NPs are observed in the TEM image, implying the highly dispersed state of Ru in the NWs. The aberration‐corrected high‐angle annular dark‐field scanning transmission electron microscopy (AC‐HAADF‐STEM) images of Ru SNC/W_18_O_49_ NWs further demonstrate that there are many bright clusters distributed on the NWs (Figure 1c). The magnified AC‐HAADF‐STEM image and corresponding 3D HAADF‐STEM surface plot image clearly show that the Ru SNC with a size of ≈1 nm is anchored on the surface of the NWs (Figures 1d,e). Moreover, as presented in high‐resolution TEM (HRTEM) images (Figure 1f), the direction consistent lattice fringes with a spacing of 0.38 nm can be assigned to the (010) planes of monoclinic W_18_O_49_, indicating that the NWs grow along the [010] direction. Energy‐dispersive X‐ray spectrum (EDS) elemental mapping presents a uniform spatial distribution of W, Ru, and O in Ru SNC/W_18_O_49_ NWs (Figure 1g). According to the EDS analysis results, the atomic ratio of Ru to W is ≈4.4:95.6 (Figure S3, Supporting Information). X‐ray diffraction (XRD) patterns of Ru SNC/W_18_O_49_ NWs and W_18_O_49_ NWs both show only the characteristic peaks of the monoclinic W_18_O_49_ phase (JCPDS, 71–2450). No diffraction peaks of Ru can be detected because of the ultra‐small size and ultralow content of Ru in the composite (Figure 1h). For comparison, W_18_O_49_ NWs and carbon black supported Ru nanoparticles (Ru NP/W_18_O_49_ NWs, Ru/C) were also prepared, respectively, whose XRD patterns exhibit distinct diffraction peaks of hexagonal close‐packed (hcp) Ru (Figures S4 and S5, Supporting Information).

**Figure 2:**
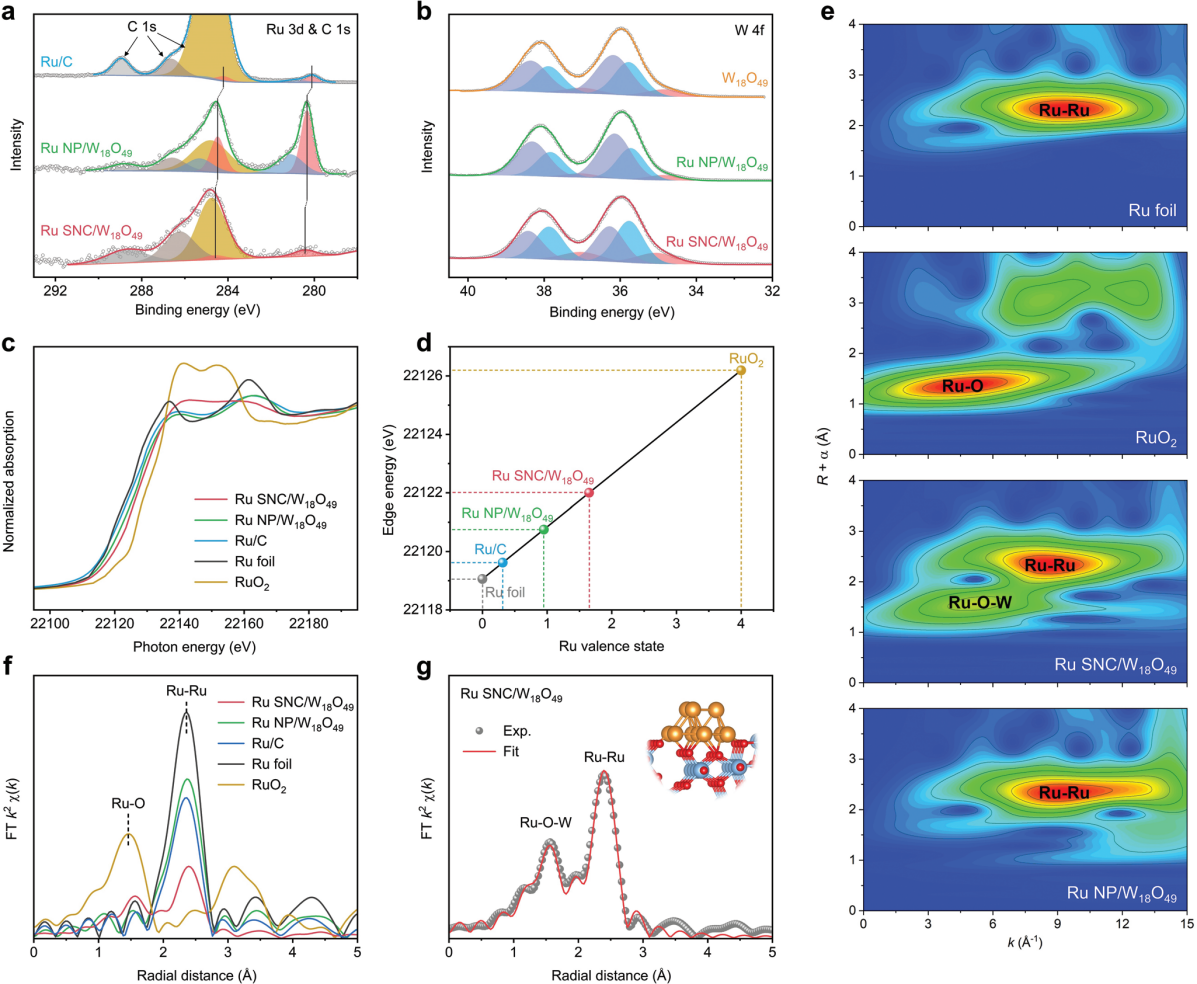
a) XPS spectra of Ru 3*d* and C 1*s* for Ru SNC/W_18_O_49_ NWs, Ru NP/W_18_O_49_ NWs, and Ru/C. b) XPS spectra of W 4*f* for Ru SNC/W_18_O_49_ NWs, Ru NP/W_18_O_49_ NWs and W_18_O_49_ NWs. c) Ru *K*‐edge XANES spectra of Ru SNC/W_18_O_49_ NWs, Ru NP/W_18_O_49_ NWs and Ru/C, with Ru foil and RuO_2_ as references. d) Relation between the Ru *K*‐edge absorption energy and valence states for Ru SNC/W_18_O_49_ NWs and reference materials. e) WT‐EXAFS and f) FT‐EXAFS spectra for different samples. g) FT‐EXAFS fit and schematic model (inset) of Ru SNC/W_18_O_49_ NWs. The orange, blue and red balls represent Ru, W, and O, respectively.

X‐ray photoelectron spectroscopy (XPS) was employed to gain insights into the surface status of the prepared materials. **Figure** **2**a shows the Ru 3*d* and C 1*s* spectra of Ru SNC/W_18_O_49_ NWs, Ru NP/W_18_O_49_ NWs, and Ru/C. The two peaks centered at 280.1 and 280.9 eV in the spectra of Ru/C are attributed to Ru 3*d*_5/2_ of Ru^0^ and Ru^4+^, respectively. In addition, the three peaks at ≈284.8, 286.6, and 288.9 eV in the spectra are assigned to C 1*s* originating from carbon species. Interestingly, Ru 3*d*_5/2_ peaks of Ru^0^ (280.4 eV) and Ru^4+^ (281.2 eV) species in Ru NP/W_18_O_49_ NWs show a shift to higher binding energy (BE) relative to that of Ru/C, revealing the electron transfer from Ru to W_18_O_49_ support. In contrast, Ru SNC/W_18_O_49_ NWs show a single Ru 3*d*_5/2_ peak at 280.6 eV, indicating the positively charged state of Ru. The W 4*f* XPS spectra of Ru SNC/W_18_O_49_ NWs, Ru NP/W_18_O_49_ NWs, and pure W_18_O_49_ NWs reveal that both W^6+^, W^5+^, and W^4+^ are presented in the samples (Figure 2b). Further fitting analysis reveals the lower average valence state of W in Ru SNC/W_18_O_49_ NWs and Ru NP/W_18_O_49_ NWs compared to pure W_18_O_49_ NWs (Table S1, Supporting Information). Particularly, the contents of W^4+^ and W^5+^ increased from 10.4% and 37.5% in W_18_O_49_ NWs to 16.4% and 44.4% in Ru SNC/W_18_O_49_ NWs, respectively, while the content of W^6+^ decreased from 52.1% to 39.2%, confirming the strong electronic interaction between Ru SNC and the W_18_O_49_ support.

X‐ray absorption near edge spectroscopy (XANES) and extended X‐ray absorption fine structure (EXAFS) were measured to further identify the electronic state and local coordination environment of different samples. Figure 2c depicts the normalized Ru *K*‐edge XANES spectra of Ru SNC/W_18_O_49_ NWs, Ru NP/W_18_O_49_ NWs, and Ru/C, with the Ru foil and RuO_2_ as references. The absorption edge of Ru/C almost overlaps with the Ru foil, which suggests the metallic state of Ru in it. In sharp contrast, the absorption edge energy of Ru SNC/W_18_O_49_ and Ru NP/W_18_O_49_ NWs both shifted to higher values, indicating the Ru atoms are positively charged due to the charge transfer between metal and W_18_O_49_ support. This phenomenon is identical to the above XPS results. Furthermore, compared with Ru NP/W_18_O_49_ NWs, the higher absorption edge energy of Ru SNC/W_18_O_49_ NWs suggests a stronger EMSI between SNCs and W_18_O_49_ compared to large NPs (Figure 2d). This conclusion is also confirmed by the W *L*_3_‐edge XANES spectra analysis (Figure S6, Supporting Information). The wavelet transforms (WT) of the Ru *K*‐edge EXAFS spectra were used to examine the coordination features of different samples (Figure 2e; Figure S7, Supporting Information). Interestingly, unlike Ru NP/W_18_O_49_ NWs and Ru/C that only show signal assigned to Ru–Ru coordination at *k* = ≈9.5 Å^−1^, the additional signal at k ≈ 4.6 Å^−1^ ascribed to Ru‐O coordination is detected in Ru SNC/W_18_O_49_ NWs. Furthermore, the Fourier transforms (FT) of the Ru *K*‐edge EXAFS spectrum and corresponding quantitative curve fittings were carried out to provide details about the local structural parameters (Figure 2f,g; Figures S8 and S9, Table S2, Supporting Information). The average coordination number of the Ru–Ru bond in Ru SNC/W_18_O_49_ NWs is only 4.9, which is obviously lower than that of Ru NP/W_18_O_49_ NWs (CN = 9.9) and Ru foil (CN = 12), corresponding to the limited atom number of SNC.^[^ ^23^ ^]^ In addition, the average coordination number of the Ru‐O bond is 2.6 in Ru SNC/W_18_O_49_ NWs, with a longer bond length (1.983 Å) than that of RuO_2_ (1.970 Å), indicating its different Ru‐O coordination environment compared to the latter. These results suggest that Ru SNCs are anchored to the W_18_O_49_ support through oxygen atoms at the interface (inset of Figure 2g).

**Figure 3:**
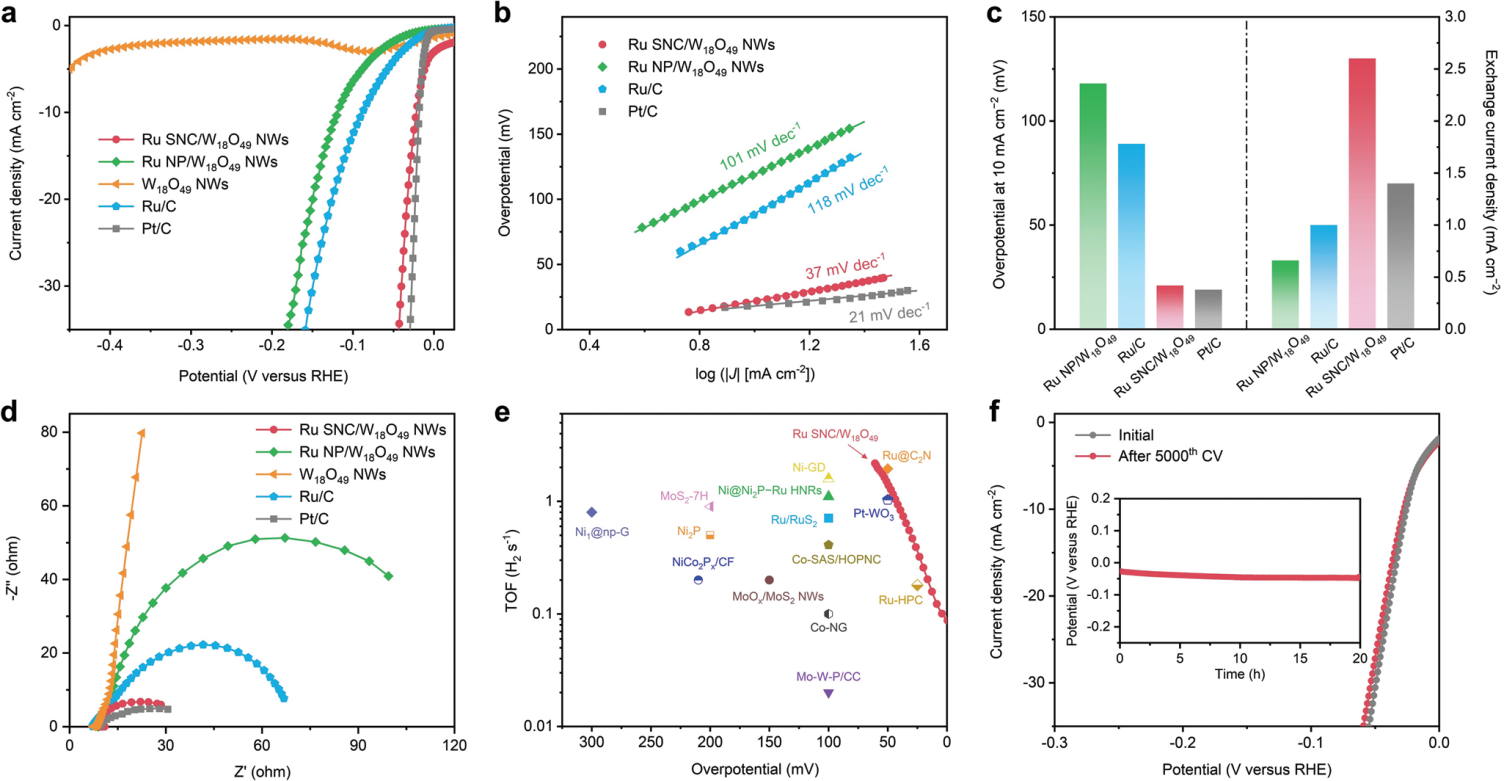
a) Polarization curves, b) Tafel slopes, c) comparison of overpotential at 10 mA cm^−2^ (left) and exchange current density (right), d) EIS Nyquist plots at the overpotential of 100 mV of Ru SNC/W_18_O_49_ NWs and comparison catalysts for HER in N_2_‐saturated 0.5 m H_2_SO_4_. e) TOF values of Ru SNC/W_18_O_49_ NWs and other recently reported representative HER electrocatalysts. f) Durability test of Ru SNC/W_18_O_49_ NWs. The polarization curves were recorded before and after 5000 potential cycles from 0.1 to −0.2 V (versus RHE). Inset shows the chronopotentiometry curve of Ru SNC/W_18_O_49_ NWs recorded at 10 mA cm^−2^.

Upon confirming the structural features of Ru SNC/W_18_O_49_ NWs, we subsequently evaluated its efficacy in electrocatalysis. As a proof of concept, we demonstrate Ru SNC/W_18_O_49_ NWs as an advanced catalyst for HER and HOR, which are two crucial reactions for realizing the future hydrogen economy. Currently, Pt is considered to be the state‐of‐the‐art electrocatalyst for HER and HOR in acidic condition, but they suffer from high cost. Therefore, the development of efficient and low‐cost electrocatalysts for HER and HOR has received extensive attention, and many advanced non‐Pt electrocatalysts have been reported in recent years.^[^ ^29^, ^30^, ^31^, ^32^ ^]^ Due to the attractive performance and desirable cost, Ru has become a highly promising candidate to replace Pt for HER and HOR, while the hydrogen binding at Ru sites (Ru‐H) is an important factor limiting its activity.^[^ ^33^, ^34^ ^]^ Considering the unique proton conductivity of tungsten oxides, which is expected to emerge unique catalytic synergies with the Ru SNCs in hydrogen electrocatalysis.^[^ ^35^, ^36^ ^]^ We first evaluated the electrocatalytic HER performance of the obtained Ru SNC/W_18_O_49_ NWs. For comparison, the HER performance of the Ru NP/W_18_O_49_ NWs, pure W_18_O_49_ NWs, Ru/C, and commercial Pt/C were also measured under the same conditions. **Figure** **3**a shows the HER polarization curves of Ru SNC/W_18_O_49_ NWs and comparison catalysts in 0.5 M H_2_SO_4_. Pure W_18_O_49_ NWs show extremely low HER current, even at a high overpotential. In contrast, Ru SNC/W_18_O_49_ NWs show an overpotential of 21 mV to achieve the current density of 10 mA cm^−2^, only 2 mV more than Pt/C (19 mV), while that of Ru/C and Ru NP/W_18_O_49_ NWs is 89 and 118 mV, respectively. Tafel slopes were evaluated to gain insight into the kinetics of the HER process. As shown in Figure 3b, a Tafel slope of 35 mV dec^−1^ was measured for Ru SA/W_18_O_49_ NWs, close to the level of Pt/C (21 mV dec^−1^), while the much larger Tafel slopes were detected for Ru/C (118 mV dec^−1^) and Ru NP/W_18_O_49_ NWs (101 mV dec^−1^). With a low Tafel slope, the HER rate of Ru SNC/W_18_O_49_ NWs will increase rapidly with increasing overpotential, leading to a competitive advantage for practical applications. By extrapolating the Tafel plots, the exchange current density of Ru SNC/W_18_O_49_ NWs was obtained (2.5 mA cm^−2^), which is higher than the most of reported Ru‐based catalysts and even Pt/C (1.4 mA cm^−2^) (Figure 3c; Table S3, Supporting Information). Furthermore, the Nyquist plots reveal that the semicircular diameter of Ru SNC/W_18_O_49_ NWs is obviously smaller than those of Ru/C and Ru NP/W_18_O_49_ NWs, suggesting its smaller charge transfer impedance (Figure 3d; Table S4, Supporting Information). The above results indicate the superior HER kinetics of Ru SNC/W_18_O_49_ NWs. Additionally, the relationship between the Ru/W ratio and HER activity was also investigated (Figures S10–S12, Supporting Information), and the 5% Ru feed ratio was confirmed to be the optimized composition among various composite catalysts. To further reveal the intrinsic electrocatalytic activity of Ru SNC/W_18_O_49_ NWs, the turnover frequency (TOF) was calculated based on the total content of Ru in the catalyst. Apparently, Ru SNC/W_18_O_49_ NWs shows higher TOF values at lower overpotentials than the most of reported catalysts, indicating its excellent intrinsic activity for HER (Figure 3e; Table S3, Supporting Information). We also evaluated the electroactive surface area (ECSA) of different electrocatalysts through the double‐layer capacitance (C_dl_) values (Figures S13 and S14, Supporting Information). Apparently, the Ru SNC/W_18_O_49_ NWs shows a higher specific activity than that of Ru NP/W_18_O_49_ NWs, W_18_O_49_ NWs, and Ru/C, further confirming its excellent electrocatalytic activity for HER. In addition to the activity, we further investigated the stability of Ru SNC/W_18_O_49_ NWs during HER. As shown in Figure 3f, the polarization curve of Ru SNC/W_18_O_49_ NWs shows negligible changes after 5000 potential cycles. The good durability of the Ru SNC/W_18_O_49_ NWs was also demonstrated by the chronopotentiometry (CP) test, in which the overpotential changed slightly during the 20 h continuously electrolyzed at a current of 10 mA cm^−2^. The HER activity of Ru SNC/W_18_O_49_ NWs in 1 M KOH was also tested, which is obviously lower than that of in 0.5 m H_2_SO_4_, probably due to the different electrocatalytic mechanism between alkaline and acidic conditions (Figure S15, Supporting Information).

**Figure 4:**
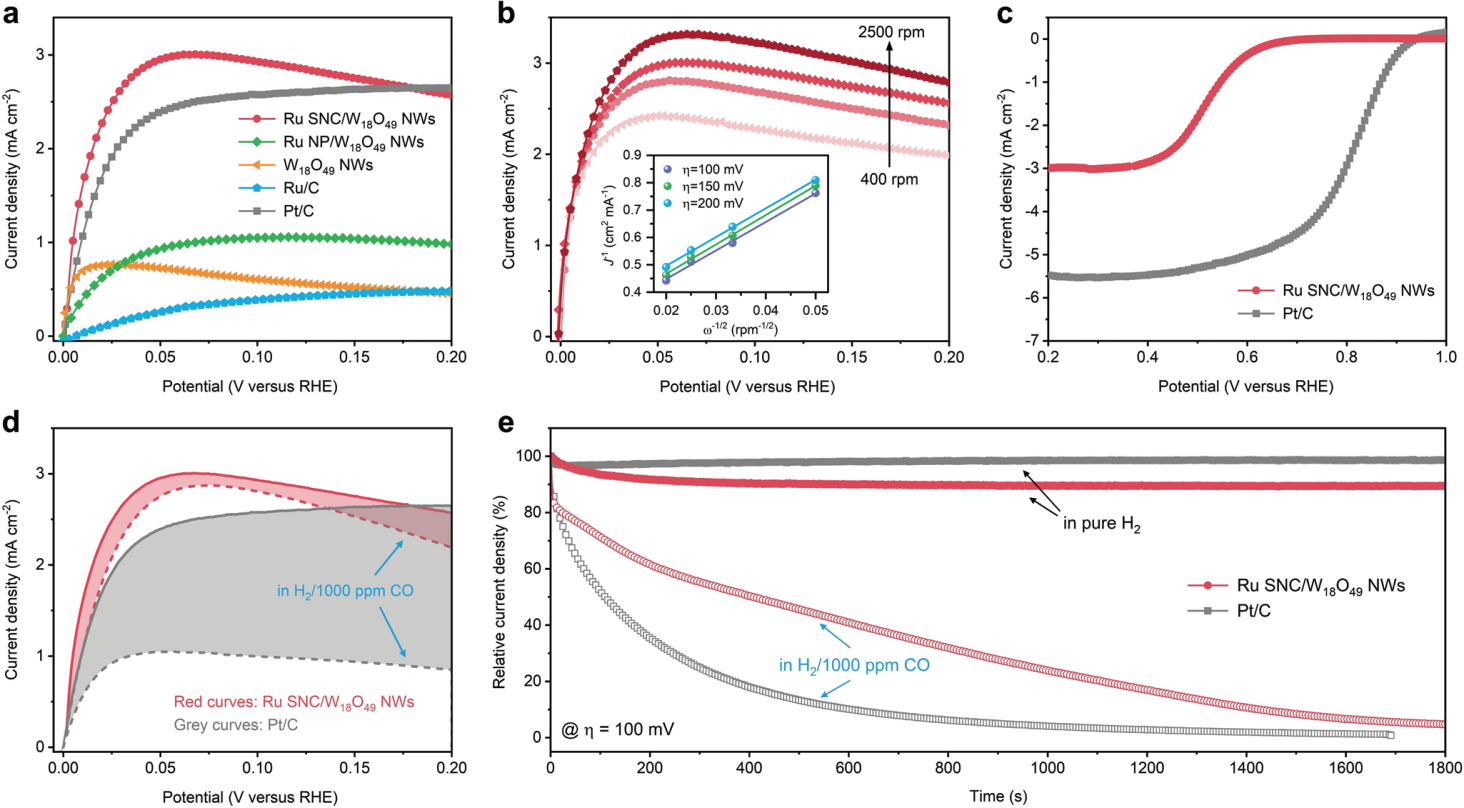
a) Polarization curves of Ru SNC/W_18_O_49_ NWs and comparison catalysts in H_2_‐saturated 0.5 m H_2_SO_4_. b) Polarization curves of Ru SNC/W_18_O_49_ NWs in H_2_‐saturated 0.5 m H_2_SO_4_ at the rotation rates of 400, 900, 1600, and 2500 rpm, respectively. Inset shows the Koutecky–Levich plots at different overpotentials. Polarization curves of Ru SNC/W_18_O_49_ NWs and Pt/C in c) O_2_‐saturated 0.5 m H_2_SO_4_ and d) H_2_/1000 ppm CO‐saturated 0.5 m H_2_SO_4_. e) Relative current‐time chronoamperometry response of Ru SNC/W_18_O_49_ NWs and Pt/C in different gas‐saturated 0.5 m H_2_SO_4_ at 0.1 V versus RHE.

Furthermore, we demonstrated Ru SNC/W_18_O_49_ NWs as an advanced catalyst for HOR, which is the anode reaction in the proton exchange membrane fuel cells (PEMFCs). The HOR catalytic performances of Ru SNC/W_18_O_49_ NWs and comparison catalysts were investigated by the rotating disk electrode (RDE) method using a standard three‐electrode system. **Figure** **4**a shows the HOR polarization curves of different catalysts in H_2_‐saturated 0.5 M H_2_SO_4_. The anode current density of Ru SNC/W_18_O_49_ NWs increases sharply with increasing potential, which is even higher than that of the state‐of‐the‐art Pt/C, and commercial Ru/C catalyst, demonstrating its excellent catalytic performance toward HOR (Figure S16 and Table S5, Supporting Information). In sharp contrast, Ru NP/W_18_O_49_ NWs, W_18_O_49_ NWs, and Ru/C show much poor HOR activity (Figure S17, Supporting Information). We also tested the HOR polarization curves of Ru SNC/W_18_O_49_ NWs at different rotating speeds (Figure 4b). The limiting current density increases along with the elevation of the rotating speed, demonstrating the H_2_ mass‐transport controlled process. The Koutecky‐Levich plots are shown in the inset of Figure 4b. Note that the LSV obtained in N_2_‐saturated electrolyte was used as the background to reduce the influence of non‐Faraday current (Figure S18, Supporting Information). An average slope of 11.1 mA^−1^ cm^2^ rpm^1/2^ is obtained, which is close to the theoretical number and indicates the current is derived from the two‐electron transfer HOR process (Figure S19, Supporting Information). Parasitic oxygen reduction reaction (ORR) at the anode due to accidental air leakage into the anode flow field, followed by transient potential jumps and severe corrosion flow field at the cathode, is currently one of the important challenges facing Pt‐based HOR catalysts.^[^ ^37^ ^]^ As shown in Figure 4c, Ru SNC/W_18_O_49_ NWs exhibit poor response to O_2_ compared to Pt/C, suggesting its better transient stability in practical applications. Moreover, the anode Pt catalyst of PEMFC is readily poisoned by impurity gas such as CO that existed in hydrogen fuel.^[^ ^38^, ^39^ ^]^ Such poisoning is caused by the preferential CO binding on metal sites, which consequently blocks the sites for hydrogen adsorption and dissociation. We, therefore, examined the HOR activities of the Ru SNC/W_18_O_49_ NWs catalyst in the presence of CO. Interestingly, the Ru SNC/W_18_O_49_ NWs showed a small decrease of HOR current even in the presence of 1000 ppm CO, indicating excellent selectivity for the HOR against CO (Figure 4d). In contrast, significant declining HOR activity on the Pt/C catalyst was detected at the same CO concentration, suggesting serious poisoning of the active sites for H_2_ oxidation by CO binding. Further chronoamperometry test revealed that Ru SNC/W_18_O_49_ NWs possess excellent stability during HOR catalysis and better tolerance to CO poisoning than that of Pt/C catalyst (Figure 4e). In addition, TEM and XPS were carried out to further characterize the Ru SNC/W_18_O_49_ NWs after the electrocatalytic test. As shown in Figure S20 (Supporting Information), Ru SNC/W_18_O_49_ NWs can well maintain their 1D structure after both the HER and HOR processes. In addition, XPS analysis revealed that the Ru 3*d* peak in Ru SNC/W_18_O_49_ NWs shifted toward high binding energy by 0.3 and 0.2 eV after HER and HOR tests, respectively. At the same time, the average valence state of W in Ru SNC/W_18_O_49_ NWs increased after the HOR test, with the content of W^6+^ increasing from an initial 39.2% to 58.5%. In contrast, the content of W^6+^ in Ru SNC/W_18_O_49_ NWs showed negligible change after HER test. The above results suggest that the surface chemical state of Ru SNC/W_18_O_49_ NWs may undergo dynamic changes during different catalytic reactions.

**Figure 5:**
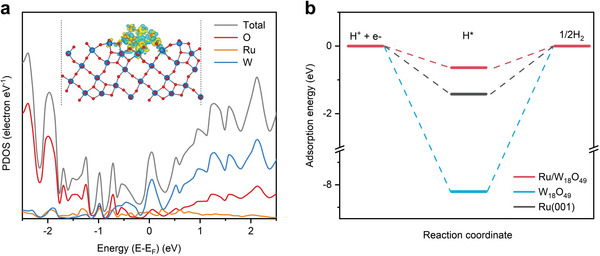
a) Projected density of states (PDOS) of Ru, W, and O atoms at Ru/W_18_O_49_. Inset shows the differential charge density distributions between Ru clusters and W_18_O_49_. b) Comparison of the hydrogen adsorption on different surface models.

Density functional theory (DFT) calculations were performed to gain insights into the mechanisms underlying the attractive electrocatalytic performance of Ru SNC/W_18_O_49_ NWs. Based on the experimental results, a composite model of Ru clusters supported on W_18_O_49_ (denoted as Ru/W_18_O_49_) was constructed. The projected density of states calculation indicates an obvious orbital overlap between Ru, W, and O for Ru/W_18_O_49_, effectively demonstrating the strong interaction between Ru and the W_18_O_49_ support (**Figure** **5**a). Moreover, the charge density difference analysis reveals the electron transfer from the Ru cluster to W_18_O_49_, which is consistent with the results of XPS and XAS (inset of Figure 5a). As proposed by Nørskov et al., the adsorption energy of hydrogen has been widely employed as a descriptor for predicting the HER/HOR performance of catalysts, which should be an optimal value for neither too strong nor too weak binding.^[^ ^40^ ^]^ The active sites of the catalyst capture the hydrogen atom by interacting with the unsaturated electron on the H 1s orbital, thus the changed electron density of Ru is expected to affect its adsorption with the hydrogen atom. Therefore, we then calculated the adsorption energy of hydrogen on Ru/W_18_O_49_, pure W_18_O_49,_ and Ru for comparison. As shown in Figure 5b, the calculated hydrogen adsorption strength of Ru/W_18_O_49_ (−0.64 eV) is weaker than that of pure Ru (−1.42 eV), which facilitates the desorption of hydrogen intermediate during HER/HOR. In addition, the hydrogen adsorption energy of the W_18_O_49_ is calculated to be −8.21 eV. Such strong hydrogen adsorption hinders the desorption of the intermediate, resulting in the poor HER/HOR catalytic activity of pure W_18_O_49_. Overall, these calculations further confirm that the electronic interaction between Ru clusters and W_18_O_49_ support and thus enables the optimized adsorption strength of hydrogen intermediate, which is consistent with the electrocatalytic performance we obtained experimentally.

## 3 Conclusion

In summary, we reported a facile one‐pot hydrothermal method for the synthesis of sub‐nanometric Ru clusters loaded W_18_O_49_ NWs as high‐performance bifunctional hydrogen electrocatalysts. Combined characterizations and theoretical calculations reveal that the electron transfer from Ru clusters to the W_18_O_49_ support optimizes the binding strength of hydrogen intermediate on Ru. With such modulation, the obtained Ru SNC/W_18_O_49_ NWs exhibit a significantly enhanced HER/HOR activity than that of compared Ru NPs, and it can even rival the activities of the state‐of the‐art Pt/C catalyst. Moreover, the excellent durability and great tolerance to CO and O_2_ impurities, coupled with the relatively low cost of Ru, makes Ru SNC/W_18_O_49_ NWs an attractive candidate to replace Pt for PEM‐based hydrogen electrocatalysis. This work demonstrates the great potential of constructing oxides‐supported sub‐nanometric clusters as high‐performance electrocatalysts.

## Conflict of Interest

The authors declare no conflict of interest.

## Supporting information

Supporting information

## Data Availability

The data that support the findings of this study are available from the corresponding author upon reasonable request.
