# Supplementary material for: Synergistic Mechanism of Sub‐Nanometric Ru Clusters Anchored on Tungsten Oxide Nanowires for High‐Efficient Bifunctional Hydrogen Electrocatalysis
Source: Adv Sci (Weinh). 2023 Jan 3;10(7):2206096. doi: 10.1002/advs.202206096 (PMC9982562; doi:10.1002/advs.202206096)
Supplement: Supplementary file 1 — Supporting information [file ADVS-10-2206096-s001.pdf]

ADVANCED SCIENCE Open Access

Supporting Information

for *Adv. Sci.*, DOI 10.1002/advs.202206096

Synergistic Mechanism of Sub-Nanometric Ru Clusters Anchored on Tungsten Oxide Nanowires for High-Efficient Bifunctional Hydrogen Electrocatalysis

*Yecan Pi, Ziming Qiu, Yi Sun, Hirofumi Ishii, Yen-Fa Liao, Xiuyun Zhang, Han-Yi Chen and Huan Pang**

Supporting Information

Synergistic Mechanism of Sub-Nanometric Ru Clusters Anchored on Tungsten Oxide Nanowires for High-Efficient Bifunctional Hydrogen Electrocatalysis

*Yecan Pi, Ziming Qiu, Yi Sun, Hirofumi Ishii, Yen-Fa Liao, Xiuyun Zhang, Han-Yi Chen, Huan Pang**

Experimental

1.1 Chemicals

Tungsten(VI) chloride (WCl_6, 99.9%) and ruthenium(III) chloride ($RuCl_3$, 99.9%) were purchased from Aladdin Industrial Corp. Ethanol (C_2H_6O, analytical reagent) and sulfuric acid (H_2SO_4, 95.0~98.0%) were purchased from Sinopharm Chemical Reagent Co. Ltd. (Shanghai, China). Nafion solution (~5 wt.% in a mixture of lower aliphatic alcohols and water) was purchased from Sigma-Aldrich. All the chemicals were used without further purification. The water (18 MΩ/cm) used in all experiments was prepared by passing through an ultra-pure purification system (Aqua Solutions).

1.2 Materials synthesis

In a typical synthesis of Ru SNC/$W_{18}O_{49}$ NWs, 0.25 mmol of WCl_6 and 100 mL of ethanol were added into a 100 mL Teflon-lined stainless-steel autoclave. The mixture was magnetically stirred for 30 min to form a clear solution. To this solution, 1 mL of ethanol solution containing 12.5 μmol of $RuCl_3$ was added, followed by stirring for another 10 min. Afterwards, the autoclave was heated at 453 K for 12 h before it was cooled to room temperature. The resultant product was separated by centrifugation, and washed with ethanol for three times. Finally, the product was dried at room temperature overnight for further use and characterization. The $Ru_x/W_{18}O_{49}$ NWs (x = 0.01, 0.03, 0.08) were obtained when the introduction amount of $RuCl_3$ were 2.5, 7.5 and 20 μmol, respectively. The pure $W_{18}O_{49}$ NWs was synthesized by following the same procedure except for the absence of $RuCl_3$ solution.

Ru NPs was synthesized as previously reported.[1] The Ru NP/$W_{18}O_{49}$ NWs and Ru/C were prepared by loading Ru NPs onto $W_{18}O_{49}$ NWs and carbon black (Vulcan XC-72), respectively.

1.3 Characterizations

Scanning electron microscopy (SEM) images and energy dispersive X-ray spectroscopy (EDS)
were taken with a Carl Zeiss GeminiSEM-300 cold field-emission scanning electron microscope operated at 15 kV. Transmission electron microscopy (TEM) was conducted on a HITACHI HT-7800 microscope at an acceleration voltage of 100 kV. High-resolution TEM (HRTEM), high-angle annular dark-field scanning TEM (HAADF-STEM) and HAADF-STEM energy dispersive X-ray spectroscopy (HAADF-STEM-EDS) were conducted on a Tecnai G2 F30 S-TWIN at an acceleration voltage of 300 kV. Partial HAADF-STEM images were taken using a TEM with probe corrector (Titan Themis Cubed G2 60-300, FEI). X-ray diffraction (XRD) patterns were collected on Bruker AXS D8 advance with Cu Kα radiation (λ=1.5418 Å). X-ray photoelectron spectra (XPS) were collected with a Thermo Fisher Scientific ESCALAB-250Xi XPS Spectrometer. Inductively coupled plasma optical emission spectroscopy (ICP-OES) measurements were performed using a simultaneous ICP spectrometer (Optima 7300 DV, Perkin Elmer) equipped with a solid-state detector. The electron paramagnetic resonance (EPR) spectra were collected using a Bruker A300-10/12 spectrometer. The Ru and W K-edge X-ray absorption spectroscopy was measured at the beamline SP12B1 of Spring-8, Hyogo Prefecture, Japan.

1.4 Electrochemical measurements

All electrochemical experiments were performed in a standard three-electrode configuration controlled by a CHI-760E electrochemical workstation (CH Instruments, Inc., Shanghai). Catalyst-coated glassy carbon electrode (GCE, Pine, 5 mm in

diameter), graphite rod and saturated calomel electrode (SCE) were used as the working, counter and reference electrode, respectively. All potentials in this study are given relative to the reversible hydrogen electrode (RHE) with iR-correction. The zero point of RHE was determined by the equilibrium potential of HER/HOR using Pt wire as working electrode in H_2-saturated electrolyte. The catalyst ink was prepared by dispersing the catalysts in water/ethanol (1:4, v/v) with 5 wt% Nafion, then the ink was drop cast on the GCE to form a catalyst thin film. The total catalyst loading on GCE was 0.85 mg cm^{-2} for Ru SNC/$W_{18}O_{49}$ NWs, Ru NP/$W_{18}O_{49}$ NWs and $W_{18}O_{49}$ NWs, 0.072 mg cm^{-2} for both Ru/C and Pt/C (20 wt%, Johnson-Matthey Inc.), respectively. The HER performance was evaluated in N_2 saturated 0.5 M H_2SO_4 solution. The HOR performance was evaluated in H_2 saturated 0.5 M H_2SO_4 solution. The sweep rate was 2 mV s^{-1} with rotation rate of 1600 rpm.

The TOF value is calculated from the equation:

$$TOF = (J \times A)/(2 \times F \times \mathrm{n})$$

J is the current density in A/cm^2. A is the area of the electrode. F is the faraday constant (a value of 96485 C/mol). n is the number of moles of the Ru or Pt that are deposited onto the electrode.

Electroactive surface area (ECSA) of different electrocatalysts were evaluated through the double-layer capacitance (C_{dl}) method.[2] The C_{dl} values were estimated by CV from 0.3 to 0.4 V with different scan rates (10, 20, 40, 60, 80 and 100 mV s^{-1}).

1.5 Theoretical computation

All the density functional theory (DFT) calculations were performed with projector-augmented wave (PAW) methods[3] within the Vienna ab initio simulation package (VASP)[4]. The exchange and correlation energies were described by the generalized gradient approximation (GGA) with PBE functional[5].The cut off energy for plane-wave basis was 400 eV. In structural relaxation, the total energy and the force on each relaxed atom were converged to 10^{-4} eV and 0.01 eV/Å, respectively. For geometry optimization and electronic calculations, the first Brillouin zone was

sampled by Γ-centered k mesh of 1×1×1 and 3×3×1, respectively. To avoid the physical interactions caused by periodic boundary conditions, the vacuum spaces were larger than 40 Å. The van der Waals interaction was also taken into consideration. The adsorption energies (ΔE_{H*}) can be defined as[6]:

$$\Delta E_{H*} = E_{basal+H} - E_{basal} - E_{H}$$

Where $E_{basal+H}$ and E_{basal} represent the total energies of Ru/$W_{18}O_{49}$ with and without adsorbed H, respectively. E_{H} is half of the H_2 energy.

Supplementary Figures: Figure S1- S20

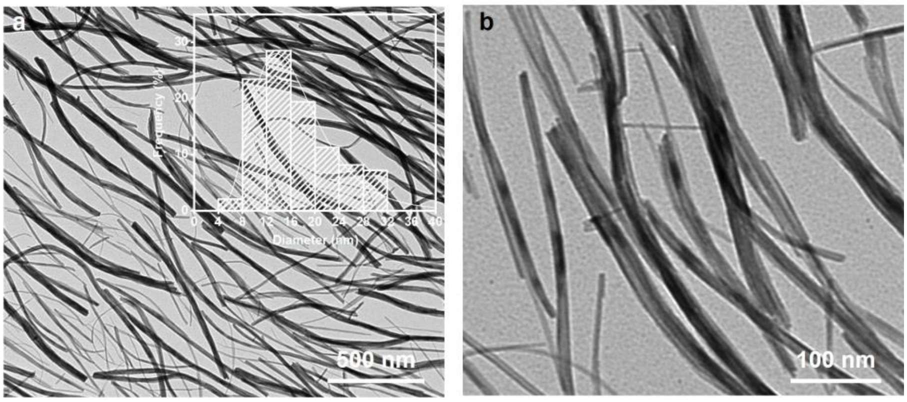

Figure S1. (a) TEM image and (b) diameter distribution of $W_{18}O_{49}$ NWs. Inset of (a) shows the corresponding diameter distribution.

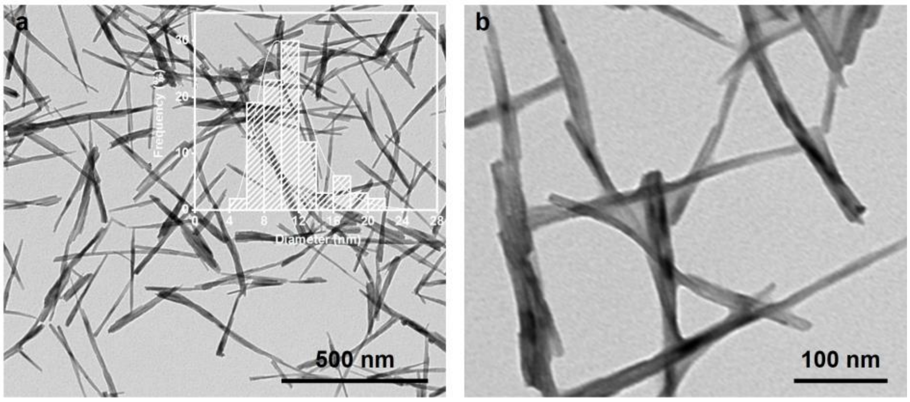

Figure S2. (a) TEM image and (b) diameter distribution of Ru SNC/$W_{18}O_{49}$ NWs. Inset of (a) shows the corresponding diameter distribution.

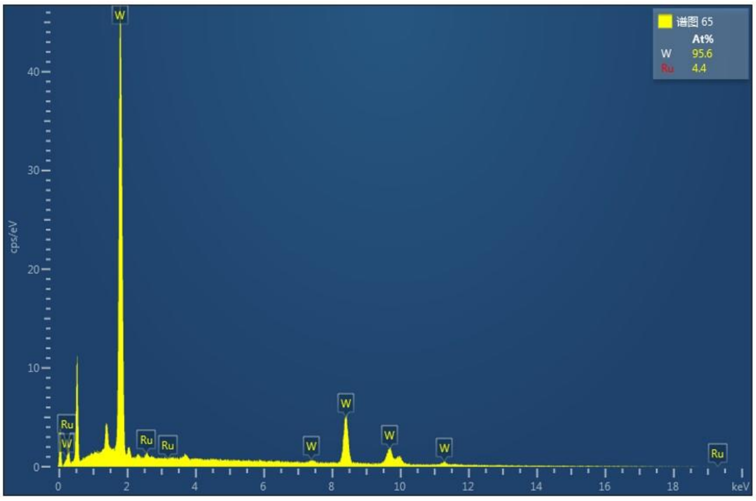

Figure S3. EDX spectrum of Ru SNC/$W_{18}O_{49}$ NWs.

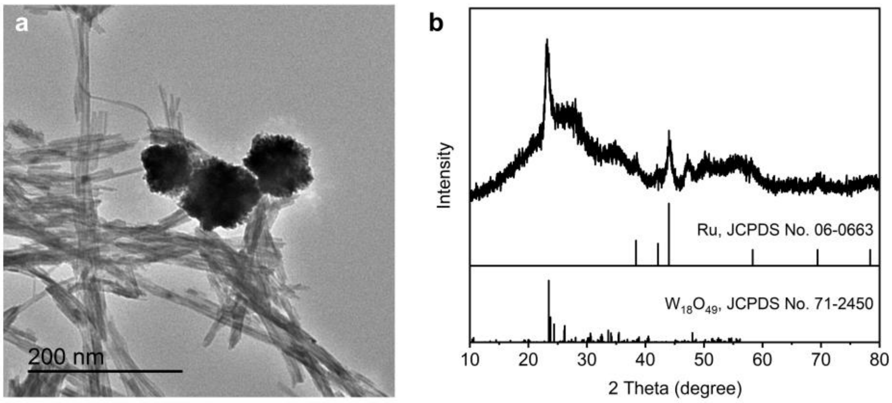

Figure S4. (a) TEM image and (b) XRD pattern of Ru NP/$W_{18}O_{49}$ NWs.

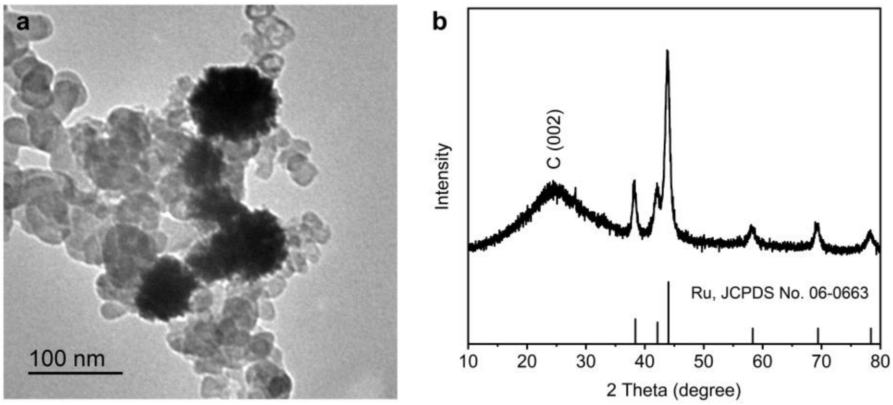

Figure S5. (a) TEM image and (b) XRD pattern of Ru/C.

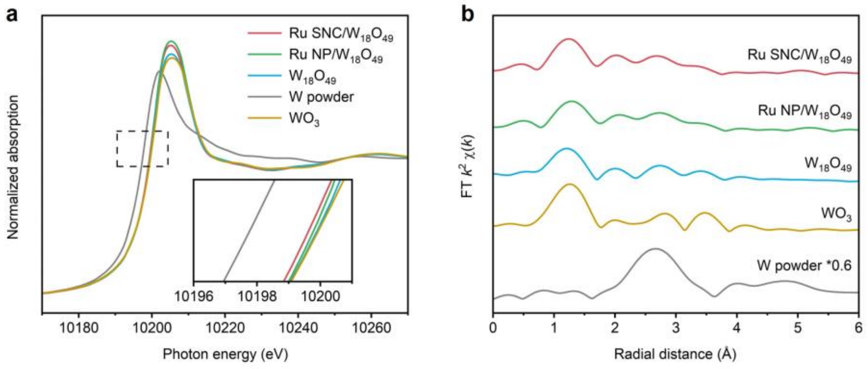

Figure S6. (a) W L_3-edge XANES and (b) FT-EXAFS spectra of Ru SNC/$W_{18}O_{49}$ NWs, Ru NP/$W_{18}O_{49}$ NWs and pure $W_{18}O_{49}$ NWs, with W powder and WO_3 as references.

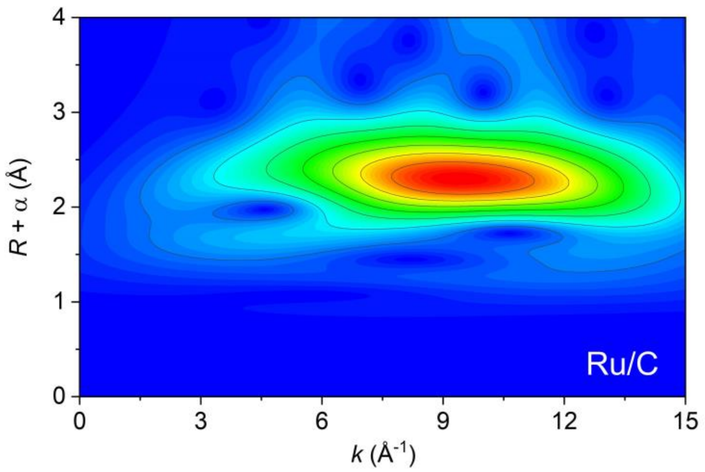

Figure S7. WT-EXAFS spectra of Ru/C.

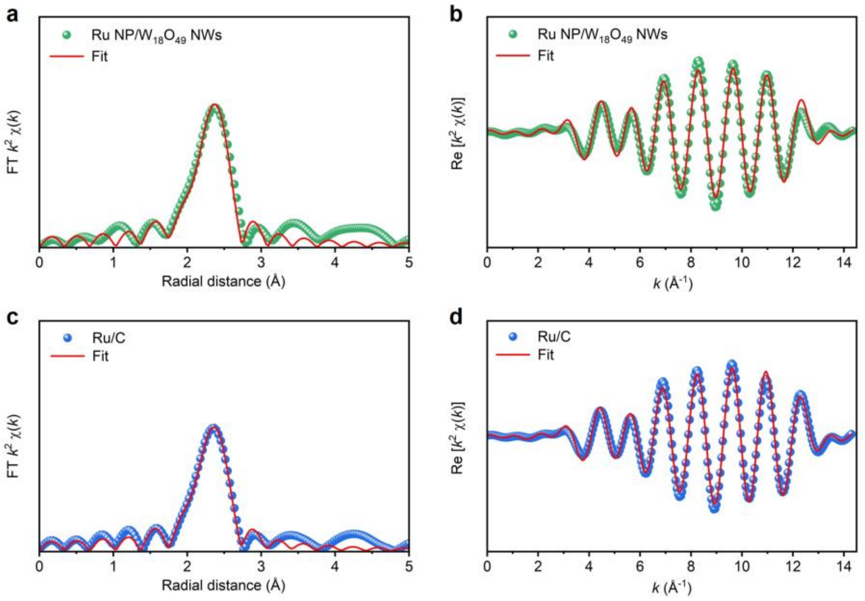

Figure S8. The fitting curve of Ru *K*-edge EXAFS spectra and $k^2\chi(k)$ oscillations of (a, b) Ru NP/$W_{18}O_{49}$ NWs and (c, d) Ru/C.

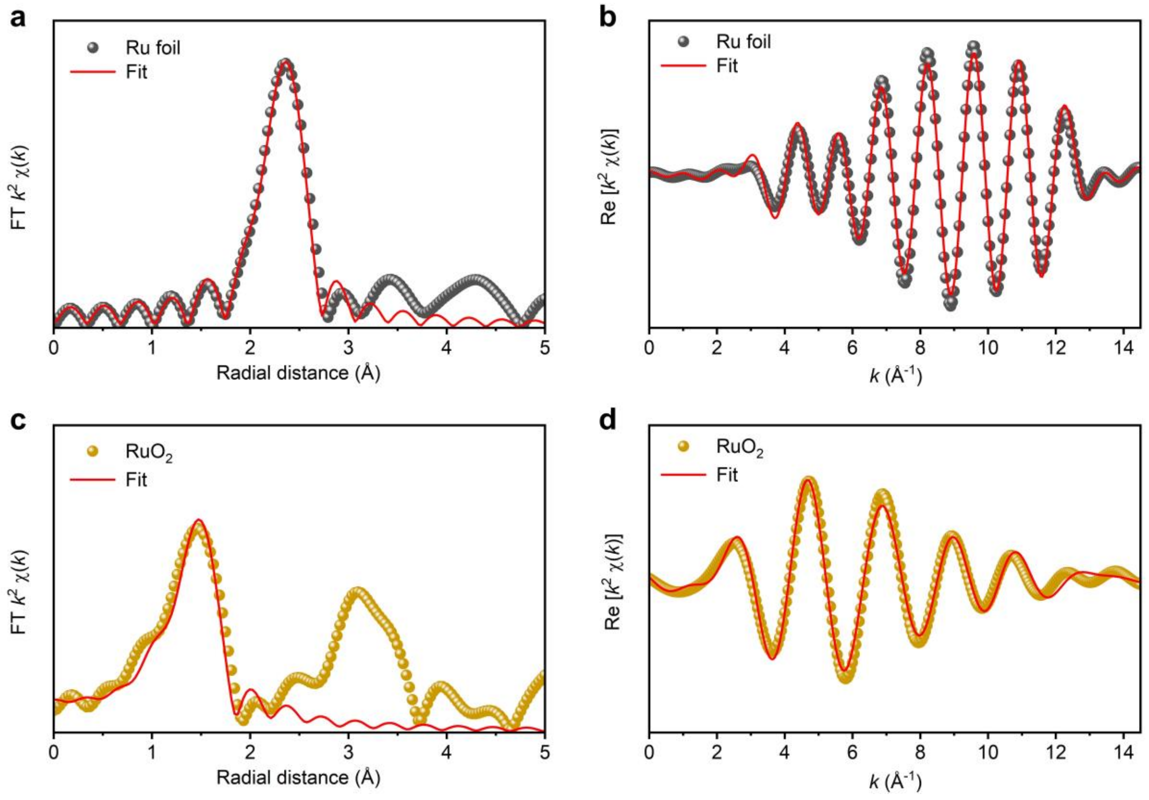

Figure S9. The fitting curve of Ru *K*-edge EXAFS spectra and $k^2\chi(k)$ oscillations of (a, b) Ru foil and (c, d) RuO_2.

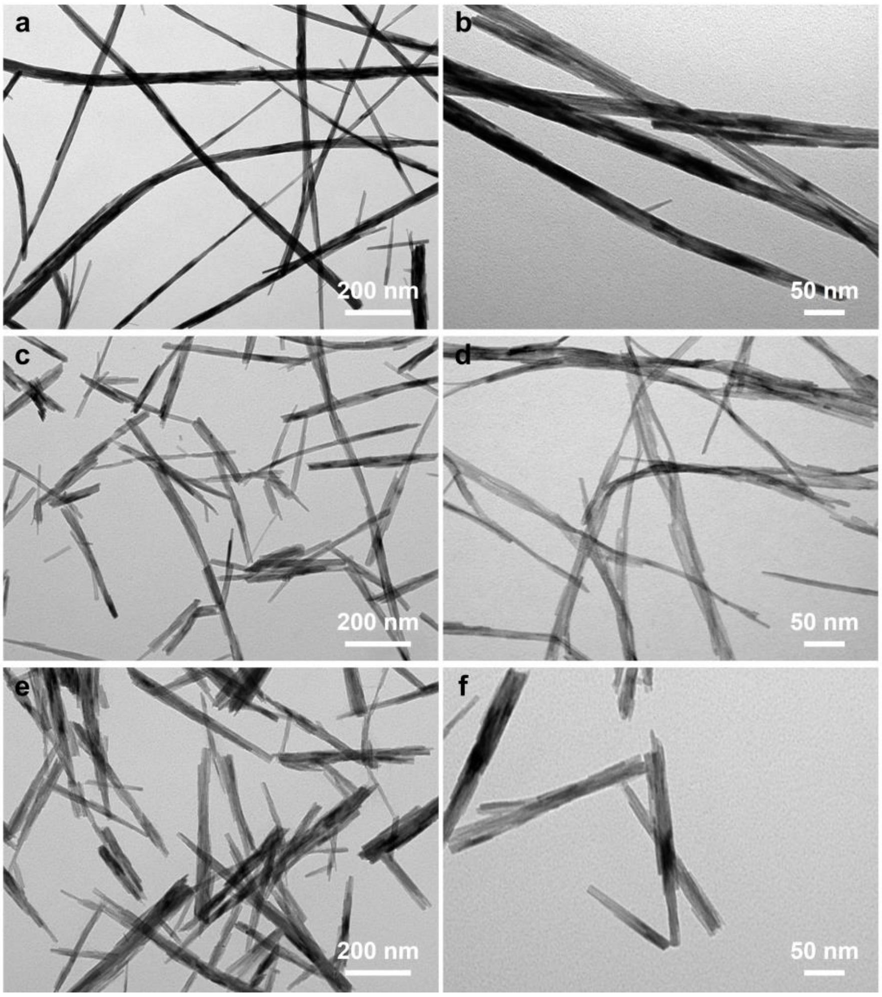

Figure S10. TEM images of (a, b) $Ru_{0.01}/W_{18}O_{49}$ NWs, (c, d) $Ru_{0.03}/W_{18}O_{49}$ NWs and (c, d) $Ru_{0.08}/W_{18}O_{49}$ NWs.

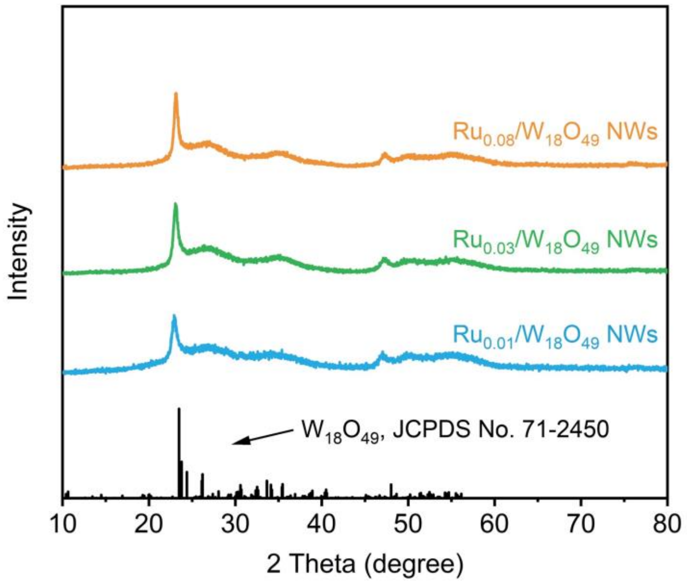

Figure S11. XRD pattens of $Ru_{0.01}/W_{18}O_{49}$ NWs, $Ru_{0.03}/W_{18}O_{49}$ NWs and $Ru_{0.08}/W_{18}O_{49}$ NWs.

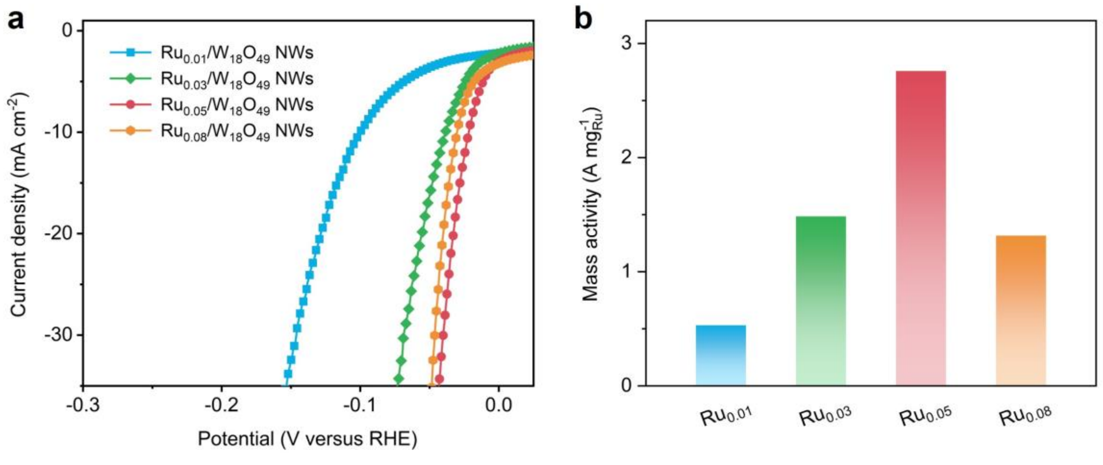

Figure S12. (a) HER polarization curves and (b) mass activities at $\eta = 50$ mV of $Ru_x/W_{18}O_{49}$ NWs.

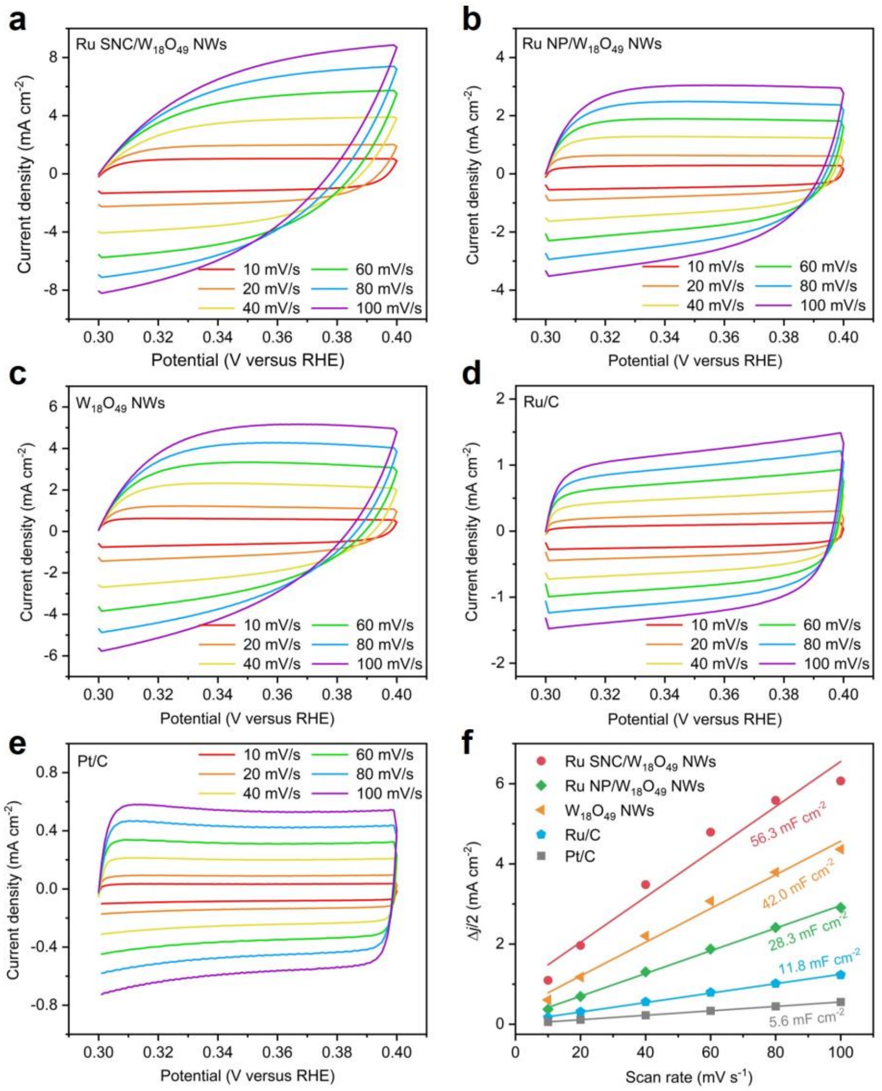

Figure S13. Cyclic voltammetry curves of (a) Ru SNC/$W_{18}O_{49}$ NWs, (b) Ru NP/$W_{18}O_{49}$ NWs, (c) $W_{18}O_{49}$ NWs, (d) Ru/C and (e) Pt/C at different scan rates. (f) Capacitive currents as a function of the scan rates.

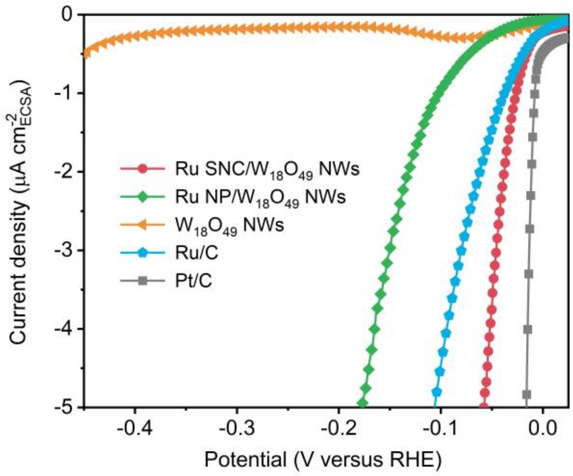

Figure S14. Electroactive surface area normalized HER polarization curves of different catalysts.

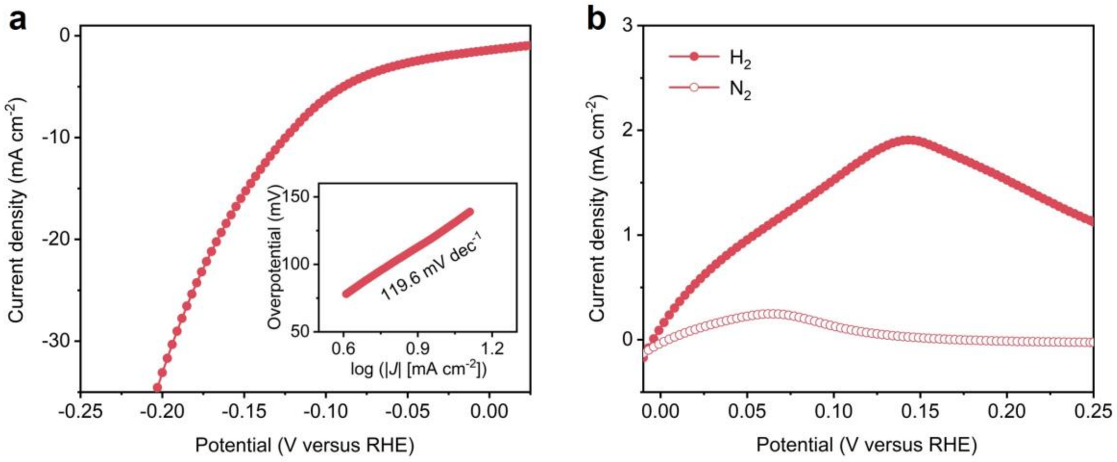

Figure S15. (a) HER polarization curve of Ru SNC/$W_{18}O_{49}$ NWs in 1 M KOH. Inset shows the corresponding Tafel plot. (b) Polarization curves of Ru SNC/$W_{18}O_{49}$ NWs in the H_2 and N_2-saturated 1 M KOH, respectively. Rotating rate: 1600 rpm.

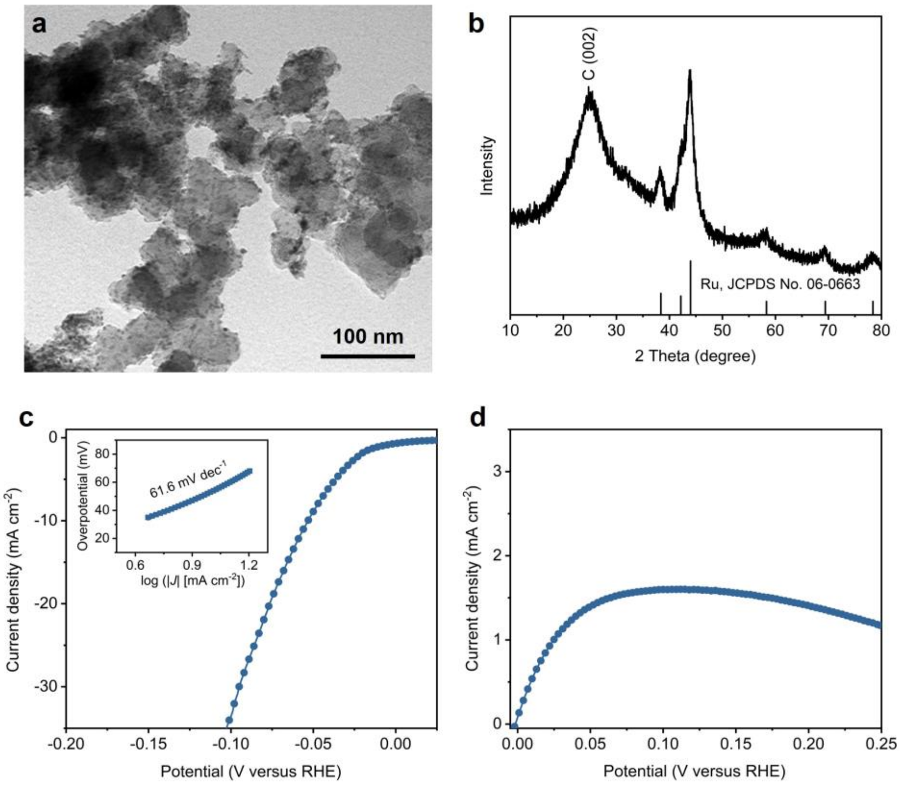

Figure S16. (a) TEM image, (b) XRD pattern, (c) HER and (d) HOR polarization curves of commercial Ru/C (20 wt% Ru on carbon from Premetek Co.). Inset in c shows the corresponding Tafel plot.

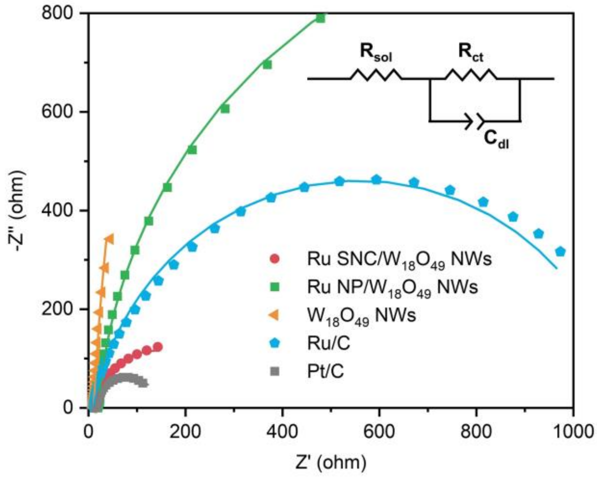

Figure S17. EIS Nyquist plots at the overpotential of 10 mV of Ru SNC/$W_{18}O_{49}$ NWs and comparison catalysts in H_2-saturated 0.5 M H_2SO_4. Rotating rate: 1600 rpm. The inset is the equivalent circuit model that contains the electrolyte resistance (R_{sol}), double layer capacitance (C_{dl}) and charge transfer resistance (R_{ct}).

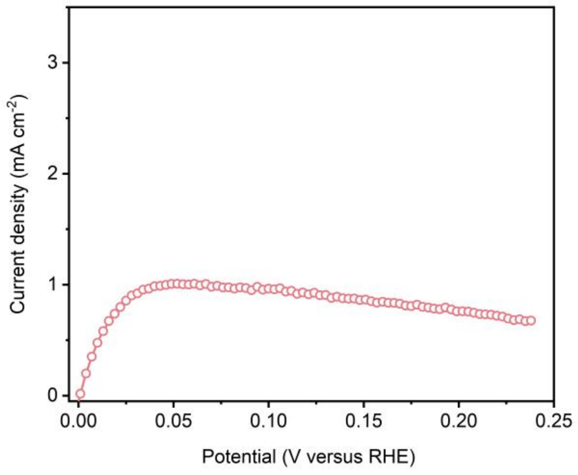

Figure S18. Polarization curves of Ru SNC/$W_{18}O_{49}$ NWs in N_2-saturated 0.5 M H_2SO_4.

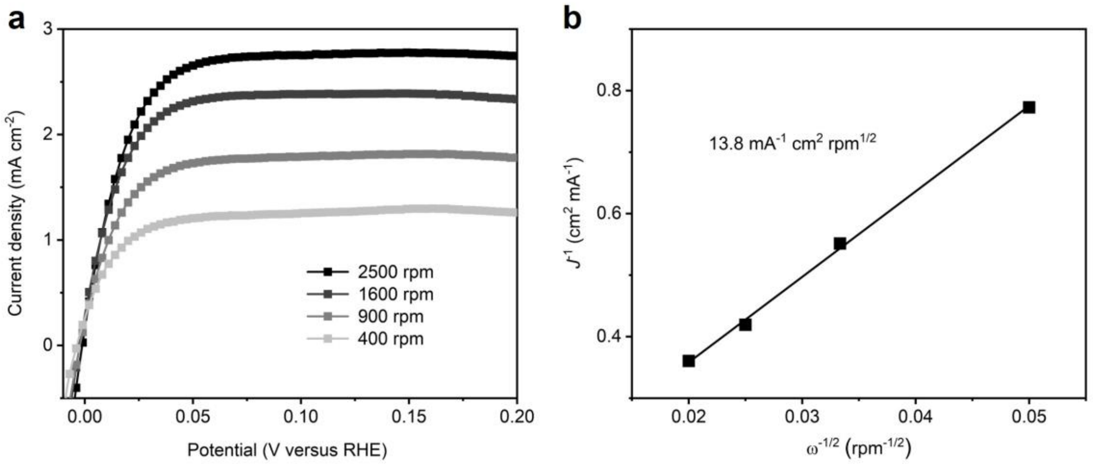

Figure S19. (a) HOR polarization curves for commercials Pt/C at various rotation speeds and (b) corresponding Koutecky-Levich plot at an overpotential of 150 mV.

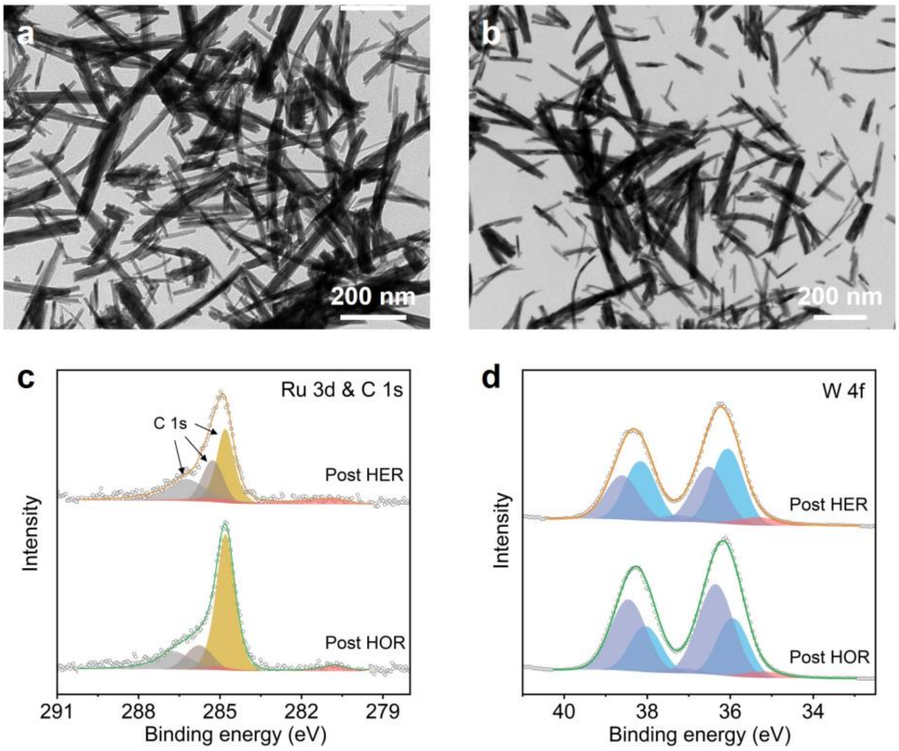

Figure S20. TEM images of Ru SNC/$W_{18}O_{49}$ NWs after the (a) HER and (b) HOR stability test. XPS spectra of (c) Ru 3*d* and C 1*s*, (d) W 4*f* for Ru SNC/$W_{18}O_{49}$ NWs after HER and HOR stability test.

Supplementary Tables: Tables S1- S5

Table S1. Summarized data from W 4*f* XPS spectra of different samples.

Sample	Valence State	Binding Energy (eV)		Concentration
		W 4$f_{7/2}$	W 4$f_{5/2}$	(at. %)
Ru SNC/$W_{18}O_{49}$ NWs	+4	35.0	37.0	16.4
	+5	35.8	37.9	44.4
	+6	36.3	38.4	39.2
Ru NP/$W_{18}O_{49}$ NWs	+4	34.9	37.0	7.0
	+5	35.7	37.8	34.5
	+6	36.1	38.2	58.5
$W_{18}O_{49}$ NWs	+4	34.8	36.9	10.4
	+5	35.8	37.9	37.5
	+6	36.2	38.3	52.1

Table S2. Summary of the Ru *K*-edge EXAFS fit parameters for the different samples. *N*, coordination number; σ^2, Debye-Waller parameters; E_0, energy shift; *R*, distance between absorber and backscatter atoms; *R*-factor represents the relative error of the fit.

Sample	Path	N	σ^2 (*10^{-3} Å^2)	ΔE_0 (eV)	R (Å)	R-factor (%)
Ru SNC /$W_{18}O_{49}$ NWs	Ru-O	2.6	0.0074±0.0043	1.5±1.3	1.983±0.018	1.5
	Ru-Ru	4.9	0.0051±0.0010		2.672±0.007	
Ru NP /$W_{18}O_{49}$ NWs	Ru-Ru	9.9	0.0040±0.0009	-3.1±1.2	2.667±0.006	3.0
Ru/C	Ru-Ru	7.3	0.0029±0.0006	-4.6±0.9	2.671±0.004	1.3
Ru foil	Ru-Ru	12	0.0031±0.0006	-5.7±0.9	2.675±0.004	1.5
RuO_2	Ru-O	6	0.0031±0.0023	1.6±2.6	1.970±0.018	3.7

Table S3. Summary of recently reported representative HER catalysts in acidic electrolyte. η is the overpotential, J_0 is the exchange current density.

Catalysts	Loading amount	η@10 mA cm^{-2} (mV)	Tafel slope (mV dec^{-1})	TOF@η (s^{-1} @mV)	J_0 (mA cm^{-2})	Ref.
Ru SNC/$W_{18}O_{49}$	18 μg_{Ru} cm^{-2}	21	35	1.47@50	2.5	This work
RuP_2@NPC	233 μg_{Ru} cm^{-2}	38	38	N/A	1.9	[7]
Ru-HPC	0.2 mg cm^{-2}	61.6	66.8	0.18@25	1.1	[8]
Ru@CN-0.16	0.26 mg cm^{-2}	126	N/A	N/A	N/A	[9]
Ru/RuS_2	0.849 mg cm^{-2}	45	24.4	0.71@100	N/A	[10]
NiRu@N–C	0.273 mg cm^{-2}	50	36	N/A	N/A	[11]
Ru-MoO_2	0.57 mg cm^{-2}	55	44	N/A	N/A	[12]
$Cu_{2-x}S$@Ru nanoplates	0.23 mg cm^{-2}	129	51	N/A	N/A	[13]
Ni@Ni_2P−Ru	0.286 mg cm^{-2}	51	35	1.1@100	0.32	[14]
Hcp-Ru@NC	0.28 mg cm^{-2}	27.5	37	1.6@25	N/A	[15]
1D-RuO_2-CN_x	171 μg_{RuO2} cm^{-2}	93	40	N/A	0.22	[16]
Ru@C_2N	0.285 mg cm^{-2}	13.5	30	1.95@50	1.9	[17]
s-RuS_2/S-rGO	176 μg_{Ru} cm^{-2}	69	64	N/A	N/A	[18]
Ru@GnP	140 μg_{Ru} cm^{-2}	13	30	N/A	N/A	[19]
C_3N_4-Ru-F	0.153 mg cm^{-2}	140	57	N/A	0.072	[20]
$Pd_3Pt_{29}Ru_{62}Te_6$	0.285 mg cm^{-2}	39	32	N/A	0.45	[21]
Ni/np-Ir	22 μg_{Ir} cm^{-2}	24	17.1	N/A	N/A	[22]
Au@Au_2Ir	20 μg_{Ir} cm^{-2}	29	N/A	N/A	N/A	[23]
L-Ag NPs	0.2 mg cm^{-2}	32	31	N/A	1.2	[24]
Pt@PCM	N/A	105	65.3	43.6@500	N/A	[25]

Pt-WO_3	0.14 mg cm^{-2}	39	32.9	1.03@50	N/A	[26]
Pt_1@Fe-N-C	63 μg_{pt} cm^{-2}	60	42	N/A	0.039	[27]
A-CoPt-NC	0.262 mg cm^{-2}	27	31	N/A	N/A	[28]
Ni-GD	N/A	86	45.8	1.59@100	0.25	[29]
Co-SAS/HOPNC	0.2 mg cm^{-2}	137	52	0.41@100	0.105	[30]
N-Mo_2C	0.357 mg cm^{-2}	99	44.5	N/A	0.1	[31]
Co-NG	0.285 mg cm^{-2}	147	82	~0.1@100	0.12	[32]
Ni_1@np-G	N/A	180	45	0.8@300	0.053	[33]
MoO_x/MoS_2 NWs	0.07 mg cm^{-2}	~300	50	0.2@150	4.5×10^{-5}	[34]
Mo-W-P/CC	4 mg cm^{-2}	138	52	0.02@100	0.29	[35]
$NiCo_2P_x$/CF	4 mg cm^{-2}	104	59.6	~0.2@210	N/A	[36]
Ni_2P	1 mg cm^{-2}	~90	46	0.5@200	3.3×10^{-5}	[37]
CoP@BCN-1	0.1 mg cm^{-2}	87	46	N/A	N/A	[38]
Mo_3P	0.5 mg cm^{-2}	69	38	4.12@200	0.28	[39]
MoS_2-cPAN	0.73 mg cm^{-2}	185	68	N/A	0.31	[40]
WC@NOC	0.209 mg cm^{-2}	51	49	N/A	2.4	[41]
MoS_2-7H	N/A	~320	~110	~0.9@200	1.9×10^{-4}	[42]
MCM@MoS_2-Ni	0.49 mg cm^{-2}	161	81	N/A	N/A	[43]
Mo_2C-Co	1 mg cm^{-2}	48	39	N/A	0.58	[44]
$MoS_{2-x}O_x$	N/A	260	67	N/A	N/A	[45]
2H-$Nb_{1.35}S_2$	N/A	123	38	N/A	0.8	[46]
CoMoP@C	0.354 mg cm^{-2}	41	49.7	N/A	1.2	[47]
Ir/$W_{18}O_{49}$	25.5 μg_{Ir} cm^{-2}	41	38	N/A	N/A	[48]

Pt-$W_{18}O_{49}$	1 mg cm^{-2}	23	30	N/A	N/A	[49]
Au@$W_{18}O_{49}$-0.70	0.17 mg cm^{-2}	79	58	N/A	N/A	[50]

Table S4. Charge transfer resistance (R_{ct}) values obtained from the EIS data fitting.

Catalysts	R_{ct} (ohm)	
	**@ -0.1 V vs. RHE**	**@ 0.01 V vs. RHE**
Ru SNC/$W_{18}O_{49}$	18.8	254.7
Ru NP/$W_{18}O_{49}$	119.6	2100
$W_{18}O_{49}$	1.0×10^{11}	1.5×10^{4}
Ru/C	64.1	1096
Pt/C	23.3	132.6

Table S5. Summary of recently reported representative HOR catalysts in acidic electrolyte. *J* is the current density, *η* is the overpotential.

Catalyst	Loading amount	Electrolyte	J at η = 25 mV (mA cm-2)	Rotating speed (rpm)	Ref.
Ru SNC/$W_{18}O_{49}$	18 μg_{Ru} cm^{-2}	0.5 M H_2SO_4	2.48	1600	This work
Ir_{NP}@Ir_{SA}-NC	5.6 μg_{Ir} cm^{-2}	0.1 M $HClO_4$	2.06	1600	[51]
IrNi/C-NH_3	6.37 μg_{IrNi} cm^{-2}	0.1 M $HClO_4$	2.22	1600	[52]
Pt/H_xWO_3/W	20 μg_{Pt} cm^{-2}	0.1 M $HClO_4$	2.03	1600	[53]
Pt-DAcPy	10 μg_{Pt} cm^{-2}	0.5 M H_2SO_4	1.18	900	[54]
Ru@RuO_2/TiO_2	100 μg_{Ru} cm^{-2}	0.1 M H_2SO_4	1.70	900	[55]
Ru@TiO_2	25 μg_{Ru} cm^{-2}	0.1 M $HClO_4$	2.12	1600	[56]
3.1 nm Ru/C	10 μg_{Ru} cm^{-2}	0.1 M H_2SO_4	0.87	2500	[57]
Polycrystalline Pt	N.A.	0.1 M H_2SO_4	2.46	1600	[58]
Pt-CTF	1.4 μg_{Pt} cm^{-2}	0.1 M $HClO_4$	1.73	2500	[59]
PtW_6O_{24}-CDs-4	10 μg_{Pt} cm^{-2}	0.1 M $HClO_4$	2.17	1600	[60]
Pd-Pd_4S/C	42.4 μg_{Pd} cm^{-2}	0.05 M H_2SO_4	1.19	1600	[61]
Rh_2P/C	6.4 μg_{Rh} cm^{-2}	0.05 M H_2SO_4	2.21	1600	[62]

References

[1] X. Kong, K. Xu, C. Zhang, J. Dai, S. Norooz Oliaee, L. Li, X. Zeng, C. Wu, Z. Peng, *ACS Catal.* **2016**, *6*, 1487.

[2] X. Lu, C. Zhao, *Nat. Commun.* **2015**, *6*, 7617.

[3] P. E. Blochl, *Phys. Rev. B* **1994**, *50*, 17953.

[4] G. Kresse, J. Furthmuller, *Comput. Mater. Sci.* **1996**, *6*, 15.

[5] J. P. Perdew, K. Burke, M. Ernzerhof, *Phys. Rev. Lett.* **1996**, *77*, 3865.

[6] J. Deng, H. Li, J. Xiao, Y. Tu, D. Deng, H. Yang, H. Tian, J. Li, P. Ren, X. Bao, *Energy Environ. Sci.* **2015**, *8*, 1594.

[7] Z. Pu, I. S. Amiinu, Z. Kou, W. Li, S. Mu, *Angew. Chem. Int. Ed.* **2017**, *56*, 11559.

[8] T. Qiu, Z. Liang, W. Guo, S. Gao, C. Qu, H. Tabassum, H. Zhang, B. Zhu, R. Zou, Y. Shao-Horn, *Nano Energy* **2019**, *58*, 1.

[9] J. Wang, Z. Wei, S. Mao, H. Li, Y. Wang, *Energy Environ. Sci.* **2018**, *11*, 800.

[10] J. Zhu, Y. Guo, F. Liu, H. Xu, L. Gong, W. Shi, D. Chen, P. Wang, Y. Yang, C. Zhang, J. Wu, J. Luo, S. Mu, *Angew. Chem. Int. Ed.* **2021**, *60*, 12328.

[11] Y. Xu, S. Yin, C. Li, K. Deng, H. Xue, X. Li, H. Wang, L. Wang, *J. Mater. Chem. A* **2018**, *6*, 1376.

[12] P. Jiang, Y. Yang, R. Shi, G. Xia, J. Chen, J. Su, Q. Chen, *J. Mater. Chem. A* **2017**, *5*, 5475.

[13] D. Yoon, J. Lee, B. Seo, B. Kim, H. Baik, S. H. Joo, K. Lee, *Small* **2017**, *13*, 1700052.

[14] Y. Liu, S. Liu, Y. Wang, Q. Zhang, L. Gu, S. Zhao, D. Xu, Y. Li, J. Bao, Z. Dai, *J. Am. Chem. Soc.* **2018**, *140*, 2731.

[15] Y. Li, L. A. Zhang, Y. Qin, F. Chu, Y. Kong, Y. Tao, Y. Li, Y. Bu, D. Ding, M. Liu, *ACS Catal.* **2018**, *8*, 5714.

[16] T. Bhowmik, M. K. Kundu, S. Barman, *ACS Appl. Mater. Interfaces* **2016**, *8*, 28678.

[17] J. Mahmood, F. Li, S.-M. Jung, M. S. Okyay, I. Ahmad, S.-J. Kim, N. Park, H. Y. Jeong, J.-B. Baek, *Nat. Nanotechnol.* **2017**, *12*, 441.

[18] J. Yu, Y. Guo, S. Miao, M. Ni, W. Zhou, Z. Shao, *ACS Appl. Mater. Interfaces* **2018**, *10*, 34098.

[19] F. Li, G.-F. Han, H.-J. Noh, I. Ahmad, I.-Y. Jeon, J.-B. Baek, *Adv. Mater.* **2018**, *30*, 1803676.

[20] Y. Peng, B. Lu, L. Chen, N. Wang, J. E. Lu, Y. Ping, S. Chen, *J. Mater. Chem. A* **2017**, *5*, 18261.

[21] S. Liu, X. Mu, W. Li, M. Lv, B. Chen, C. Chen, S. Mu, *Nano Energy* **2019**, *61*, 346.

[22] Y. Yu, K. Jiang, M. Luo, Y. Zhao, J. Lan, M. Peng, F. M. F. de Groot, Y. Tan, *ACS Nano* **2021**, *15*, 5333.

[23] H. Wang, Z.-n. Chen, D. Wu, M. Cao, F. Sun, H. Zhang, H. You, W. Zhuang, R. Cao, *J. Am. Chem. Soc.* **2021**, *143*, 4639.

[24] Z. Li, J.-Y. Fu, Y. Feng, C.-K. Dong, H. Liu, X.-W. Du, *Nat. Catal.* **2019**, *2*, 1107-1114.

[25] H. Zhang, P. An, W. Zhou, B. Y. Guan, P. Zhang, J. Dong, X. W. Lou, *Sci. Adv.* **2018**, *4*, eaao6657.

[26] C. Xie, W. Chen, S. Du, D. Yan, Y. Zhang, J. Chen, B. Liu, S. Wang, *Nano Energy* **2020**, *71*, 104653.

[27] X. Zeng, J. Shui, X. Liu, Q. Liu, Y. Li, J. Shang, L. Zheng, R. Yu, *Adv. Energy Mater.* **2018**, *8*, 1701345.

[28] L. Zhang, Y. Jia, H. Liu, L. Zhuang, X. Yan, C. Lang, X. Wang, D. Yang, K. Huang, S. Feng, X. Yao, *Angew. Chem. Int. Ed.* **2019**, *58*, 9404.

[29] Y. Xue, B. Huang, Y. Yi, Y. Guo, Z. Zuo, Y. Li, Z. Jia, H. Liu, Y. Li, *Nat. Commun.* **2018**, *9*, 1460.

[30] T. Sun, S. Zhao, W. Chen, D. Zhai, J. Dong, Y. Wang, S. Zhang, A. Han, L. Gu, R. Yu, X. Wen, H. Ren, L. Xu, C. Chen, Q. Peng, D. Wang, Y. Li, *Proc. Natl. Acad. Sci.* **2018**, *115*, 12692.

[31] J. Jia, T. Xiong, L. Zhao, F. Wang, H. Liu, R. Hu, J. Zhou, W. Zhou, S. Chen, *ACS Nano* **2017**, *11*, 12509.

[32] H. Fei, J. Dong, M. J. Arellano-Jiménez, G. Ye, N. Dong Kim, E. L. G. Samuel, Z. Peng, Z. Zhu, F. Qin, J. Bao, M. J. Yacaman, P. M. Ajayan, D. Chen, J. M. Tour, *Nat. Commun.* **2015**, *6*, 8668.

[33] H.-J. Qiu, Y. Ito, W. Cong, Y. Tan, P. Liu, A. Hirata, T. Fujita, Z. Tang, M. Chen, *Angew. Chem. Int. Ed.* **2015**, *54*, 14031.

[34] D. R. Cummins, U. Martinez, A. Sherehiy, R. Kappera, A. Martinez-Garcia, R. K. Schulze, J. Jasinski, J. Zhang, R. K. Gupta, J. Lou, M. Chhowalla, G. Sumanasekera, A. D. Mohite, M. K. Sunkara, G. Gupta, *Nat. Commun.* **2016**, *7*, 11857.

[35] X.-D. Wang, Y.-F. Xu, H.-S. Rao, W.-J. Xu, H.-Y. Chen, W.-X. Zhang, D.-B. Kuang, C.-Y. Su, *Energy Environ. Sci.* **2016**, *9*, 1468.

[36] R. Zhang, X. Wang, S. Yu, T. Wen, X. Zhu, F. Yang, X. Sun, X. Wang, W. Hu, *Adv. Mater.* **2017**, *29*, 1605502.

[37] E. J. Popczun, J. R. McKone, C. G. Read, A. J. Biacchi, A. M. Wiltrout, N. S. Lewis, R. E. Schaak, *J. Am. Chem. Soc.* **2013**, *135*, 9267.

[38] H. Tabassum, W. Guo, W. Meng, A. Mahmood, R. Zhao, Q. Wang, R. Zou, *Adv. Energy Mater.* **2017**, *7*, 1601671.

[39] A. Kondori, M. Esmaeilirad, A. Baskin, B. Song, J. Wei, W. Chen, C. U. Segre, R. Shahbazian-Yassar, D. Prendergast, M. Asadi, *Adv. Energy Mater.* **2019**, *9*, 1900516.

[40] T. S. Zeleke, M.-C. Tsai, M. A. Weret, C.-J. Huang, M. K. Birhanu, T.-C. Liu, C.-P. Huang, Y.-L. Soo, Y.-W. Yang, W.-N. Su, B.-J. Hwang, *ACS Nano* **2019**, *13*, 6720.

[41] Y.-T. Xu, X. Xiao, Z.-M. Ye, S. Zhao, R. Shen, C.-T. He, J.-P. Zhang, Y. Li, X.-M. Chen, *J. Am. Chem. Soc.* **2017**, *139*, 5285.

[42] L. Li, Z. Qin, L. Ries, S. Hong, T. Michel, J. Yang, C. Salameh, M. Bechelany, P. Miele, D. Kaplan, M. Chhowalla, D. Voiry, *ACS Nano* **2019**, *13*, 6824.

[43] H. Zhang, L. Yu, T. Chen, W. Zhou, X. W. Lou, *Adv. Funct. Mater.* **2018**, *28*, 1807086.

[44] X. Zang, W. Chen, X. Zou, J. N. Hohman, L. Yang, B. Li, M. Wei, C. Zhu, J. Liang, M. Sanghadasa, J. Gu, L. Lin, *Adv. Mater.* **2018**, *30*, 1805188.

[45] J. Pető, T. Ollár, P. Vancsó, Z. I. Popov, G. Z. Magda, G. Dobrik, C. Hwang, P. B. Sorokin, L. Tapasztó, *Nat. Chem.* **2018**, *10*, 1246.

[46] J. Yang, A. R. Mohmad, Y. Wang, R. Fullon, X. Song, F. Zhao, I. Bozkurt, M. Augustin, E. J. G. Santos, H. S. Shin, W. Zhang, D. Voiry, H. Y. Jeong, M. Chhowalla, *Nat. Mater.* **2019**, *18*, 1309.

[47] Y.-Y. Ma, C.-X. Wu, X.-J. Feng, H.-Q. Tan, L.-K. Yan, Y. Liu, Z.-H. Kang, E.-B. Wang, Y.-G. Li, *Energy Environ. Sci.* **2017**, *10*, 788.

[48] C. Peng, W. Zhao, Z. Kuang, J. T. Miller, H. Chen, *Appl. Catal. A: Gen.* **2021**, *623*, 118293.

[49] W. X. Li, Z. Y. Liu, S. C. Yang, J. N. Wu, L. Sun, E. G. Ma, H. G. Yang, X. Guo, *Sci. China Mater.* **2022**, DOI: 10.1007/s40843-022-2258-3.

[50] Y. Yu, Z. Zhou, X. Song, X. Song, Z. Zhang, C. Jing, *Inorg. Chem. Front.* **2022**, *9*, 4785.

[51] X. Yang, Y. Wang, X. Wang, B. Mei, E. Luo, Y. Li, Q. Meng, Z. Jin, Z. Jiang, C. Liu, J. Ge, W. Xing, *Angew. Chem. Int. Ed.* **2021**, *60*, 26177.

[52] W. Zhang, L. Li, W. Ding, S. Chen, H. Wang, Z. Wei, *J. Mater. Chem. A* **2014**, *2*, 10098.

[53] S.-M. Jung, S.-W. Yun, J.-H. Kim, S.-H. You, J. Park, S. Lee, S. H. Chang, S. C. Chae, S. H. Joo, Y. Jung, J. Lee, J. Son, J. Snyder, V. Stamenkovic, N. M. Markovic, Y.-T. Kim, *Nat. Catal.* **2020**, *3*, 639.

[54] T. Wang, Z.-X. Chen, S. Yu, T. Sheng, H.-B. Ma, L.-N. Chen, M. Rauf, H.-P. Xia, Z.-Y. Zhou, S.-G. Sun, *Energy Environ. Sci.* **2018**, *11*, 166.

[55] T. Wang, L.-Y. Li, L.-N. Chen, T. Sheng, L. Chen, Y.-C. Wang, P. Zhang, Y.-H. Hong, J. Ye, W.-F. Lin, Q. Zhang, P. Zhang, G. Fu, N. Tian, S.-G. Sun, Z.-Y. Zhou, *J. Am. Chem. Soc.* **2022**, *144*, 9292.

[56] Y. Zhou, Z. Xie, J. Jiang, J. Wang, X. Song, Q. He, W. Ding, Z. Wei, *Nat. Catal.* **2020**, *3*, 454.

[57] J. Ohyama, T. Sato, Y. Yamamoto, S. Arai, A. Satsuma, *J. Am. Chem. Soc.* **2013**, *135*, 8016.

[58] W. Sheng, Z. Zhuang, M. Gao, J. Zheng, J. G. Chen, Y. Yan, *Nat. Commun.* **2015**, *6*, 5848.

[59] R. Kamai, K. Kamiya, K. Hashimoto, S. Nakanishi, *Angew. Chem. Int. Ed.* **2016**, *55*, 13184.

[60] Y. Zhou, F. Yu, Z. Lang, H. Nie, Z. Wang, M. Shao, Y. Liu, H. Tan, Y. Li, Z. Kang, *Chem. Eng. J.* **2021**, *426*, 130709.

[61] L. Su, Y. Zhao, Y. Jin, Z. Liu, H. Cui, W. Luo, *Adv. Funct. Mater.* **2022**, *32*, 2113047.

[62] F. Yang, X. Bao, D. Gong, L. Su, G. Cheng, S. Chen, W. Luo, *ChemElectroChem* **2019**, *6*, 1990.
